# The network is more important than the node: stereo-EEG evidence of neurocognitive networks in epilepsy

**DOI:** 10.3389/fnetp.2024.1424004

**Published:** 2024-07-24

**Authors:** Nicholas W. G. Murray, Anthony C. Kneebone, Petra L. Graham, Chong H. Wong, Greg Savage, Lisa Gillinder, Michael W. K. Fong

**Affiliations:** ^1^ School of Psychological Sciences, Macquarie University, Sydney, Australia; ^2^ Westmead Comprehensive Epilepsy Centre, The University of Sydney, Sydney, Australia; ^3^ School of Psychology, University of Queensland, Brisbane, Australia; ^4^ Department of Neurology and Stroke, Flinders Medical Centre, Adelaide, Australia; ^5^ School of Mathematical and Physical Sciences, Macquarie University, Sydney, Australia; ^6^ Advanced Epilepsy Unit, The Mater Hospital, Brisbane, Australia; ^7^ Comprehensive Epilepsy Center, Department of Neurology, Yale University School of Medicine, New Haven, CT, United States

**Keywords:** epilepsy networks, interictal activity, stereoelectroencephalography, neuropsychology, temporal lobe epilepsy, frontal lobe epilepsy

## Abstract

**Introduction:**

Neuropsychological assessment forms an integral part of the presurgical evaluation for patients with medically refractory focal epilepsy. Our understanding of cognitive impairment in epilepsy is based on seminal lesional studies that have demonstrated important structure-function relationships within the brain. However, a growing body of literature demonstrating heterogeneity in the cognitive profiles of patients with focal epilepsy (e.g., temporal lobe epilepsy; TLE) has led researchers to speculate that cognition may be impacted by regions outside the seizure onset zone, such as those involved in the interictal or “irritative” network.

**Methods:**

Neuropsychological data from 48 patients who underwent stereoelectroencephalography (SEEG) monitoring between 2012 and 2023 were reviewed. Patients were categorized based on the site of seizure onset, as well as their irritative network, to determine the impact of wider network activity on cognition. Neuropsychological data were compared with normative standards (i.e., z = 0), and between groups.

**Results:**

There were very few distinguishing cognitive features between patients when categorized based purely on the seizure onset zone (i.e., frontal lobe vs. temporal lobe epilepsy). In contrast, patients with localized irritative networks (i.e., frontal *or* temporal interictal epileptiform discharges [IEDs]) demonstrated more circumscribed profiles of impairment compared with those demonstrating wider irritative networks (i.e., frontotemporal IEDs). Furthermore, the directionality of propagation within the irritative network was found to influence the manifestations of cognitive impairment.

**Discussion:**

The findings suggest that neuropsychological assessment is sensitive to network activity beyond the site of seizure onset. As such, an overly focal interpretation may not accurately reflect the distribution of the underlying pathology. This has important implications for presurgical work-up in epilepsy, as well as subsequent surgical outcomes.

## 1 Introduction

Neuropsychological assessment is an essential presurgical investigation for patients undergoing the workup for medically-refractory focal epilepsy. Advances in structural and functional neuroimaging have reduced the reliance on cognitive testing to localize seizure foci (Baxendale, 2018), however, neuropsychological assessment remains a fundamental measure of seizure-related cortical dysfunction. The accurate localization of cortical dysfunction has significant implications for surgical candidacy and is highly valuable when counselling patients as to the risks of resective or ablative approaches.

**FIGURE 1:**
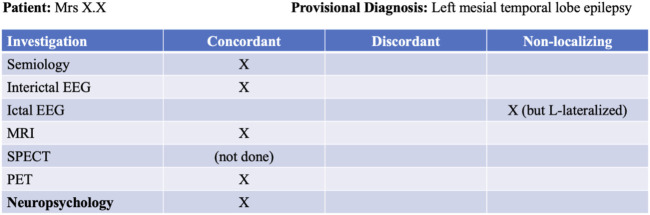
Example of a presurgical evaluation table used to chart consensus among non-invasive investigations.

Our foundational knowledge of cognitive impairment in epilepsy is based on seminal lesional studies that have demonstrated important structure-function relationships within the brain (e.g., Penfield, 1939; Scoville and Milner, 1957; Penfield and Milner, 1958). However, the understanding of cognition in epilepsy has since evolved to acknowledge the important impact of respective seizure networks (Rayner and Tailby, 2017). Cognitive impairment has historically been interpreted as an epilepsy or seizure-related indication of focal dysfunction—often limited to a specific region or lobe within the brain. In the context of a patient’s presurgical evaluation, the findings from neuropsychological assessment may be labelled as either *concordant* or *discordant* with the provisional hypothesis (e.g., see Figure 1). While this would be appropriate if cognitive impairment was strictly indicative of the epileptogenic zone (EZ), there is a growing body of literature arguing against a dichotomous approach to neuropsychology in epilepsy (Stretton and Thompson, 2012; Rayner and Tailby, 2017). As such, while the findings may be discordant with the site of seizure onset, they may still provide important insight into regions beyond this area that are involved within the patient’s seizure network, rather than being determined by a single node within that network.

Speculation regarding the localizing value of neuropsychological assessment has come from research demonstrating significant heterogeneity in cognitive deficits within focal epilepsy subtypes—namely with temporal lobe epilepsy (TLE) patients displaying deficits in executive functioning (Agah et al., 2017; Ren et al., 2020) and frontal lobe epilepsy (FLE) patients exhibiting impaired memory and language (Centeno et al., 2010). One explanation for these mixed findings is that seizure activity is often present beyond the site of seizure onset, ultimately involving distal structures, resulting in more widespread cognitive impairment (Stretton and Thompson, 2012; Besson et al., 2014; Dinkelacker et al., 2016).

**FIGURE 2:**
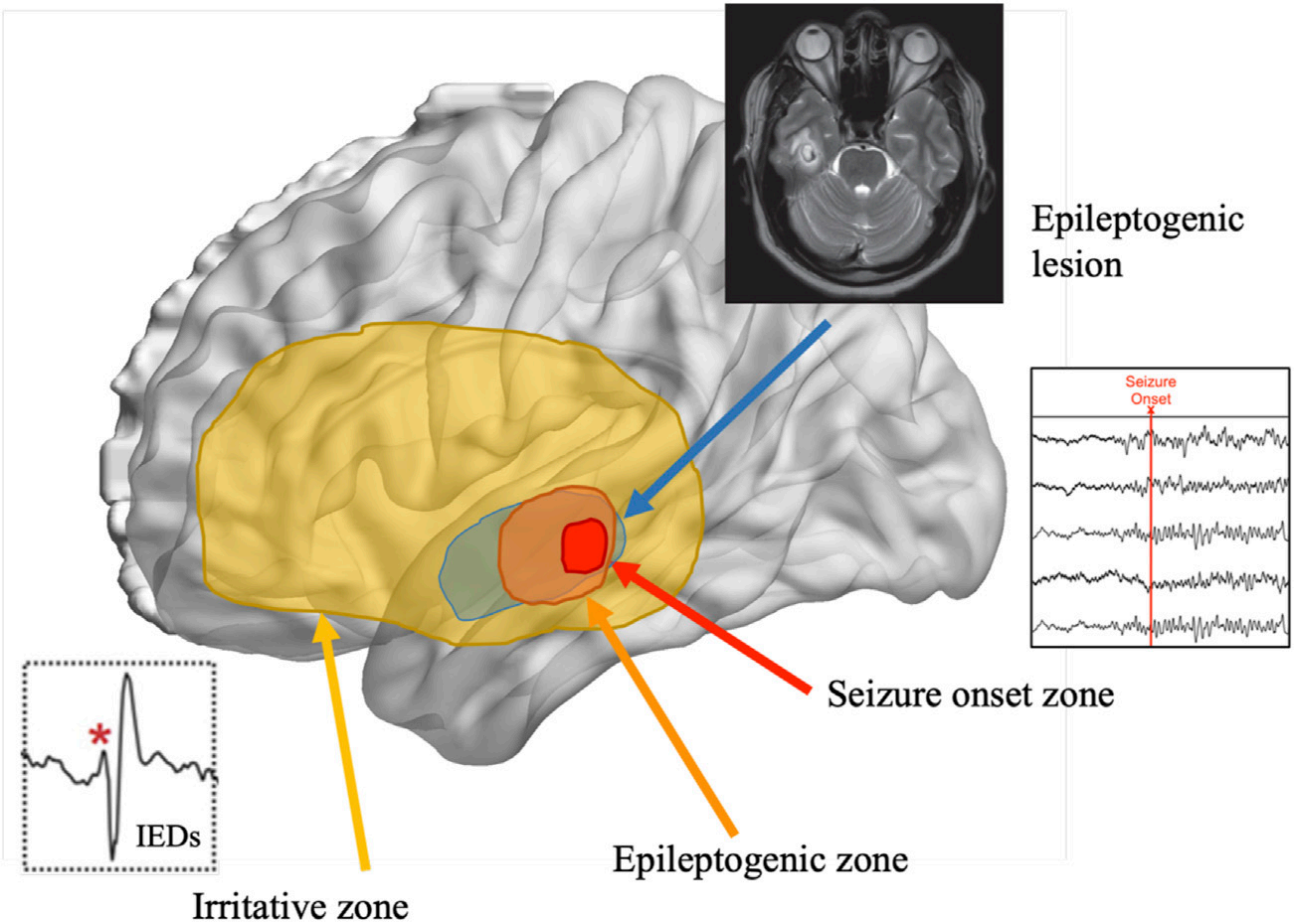
Representation of the overlapping cortical zones in epilepsy and their relation to the epileptogenic zone. *Note.* Adapted from Tamilia et al., 2017.

Over the past several decades, our understanding of epilepsy has evolved to consider various overlapping cortical zones that ultimately define an individual’s seizures (see Figure 2). The EZ is the area of cortex involved in seizure onset and initial propagation, whose removal is necessary for seizure freedom (Lüders et al., 1993; 2006). The EZ can extend beyond the primary seizure onset zone and can only be confirmed, by definition, after surgery has rendered the patient seizure free (Jehi, 2018). The irritative zone (IZ; Bettus et al., 2011) is a broader region beyond the EZ that generates epileptic activity in the form of interictal epileptiform discharges (IEDs; excluding focal slowing), which occur in the absence of any observable behavioral change (Glennon et al., 2016). Importantly, accurate identification of these zones often requires the added spatiotemporal precision of intracranial depth electrodes (i.e., stereoelectroencephalography [SEEG]; Henin et al., 2021), as it is often undetectable via scalp-EEG (Spencer et al., 1998; Bettus et al., 2011).

In a study investigating executive dysfunction in TLE patients, Reyes et al. (2018) suggested that the propagation of IEDs to ipsilateral frontal regions may underlie poor performance on executive measures. Indeed, several studies have demonstrated that widespread IEDs can impact the connectivity of frontotemporal networks (Fahoum et al., 2012; Maccotta et al., 2013; Shamshiri et al., 2016; Bou Assi et al., 2020). This has been proposed as an explanation for the high incidence of widespread cognitive impairment in TLE patients, including domains outside of those typically attributed to temporal lobe function (Reyes et al., 2020). Localization of activity beyond the suspected site of seizure onset has important implications for presurgical planning and subsequent surgical success, as recurrence of seizure after surgery is often associated with residual epileptogenic tissue in ipsilateral neighboring structures (Ryvlin and Kahane, 2005; Elwan et al., 2013).

While the impact of more frequent and/or widespread IEDs has been investigated in TLE, the specific pattern of interictal networks has seldom been explored (e.g., IED networks involving frontal regions vs. parietal regions). Often, patients with extratemporal IEDs are categorized into one group based on the site of seizure onset (e.g., TLE), regardless of the network involvement. Furthermore, little consideration has been given to the interplay between the site of onset and the subsequent propagation pattern with cognition or the directionality of propagation within the network (i.e., temporal lobe onset with frontal propagation vs. frontal lobe onset with temporal propagation). To date, no study has investigated the impact of network interictal activity on cognitive impairment in an exclusively SEEG sample—an important distinction given interictal activity can be difficult to detect and localize via scalp-EEG.

The current study aimed to investigate the cognitive profiles of patients when categorized based on the seizure onset zone (a single node) or the irritative zone (broader epileptic network). It extends on previous research by specifically investigating patients who had progressed to SEEG monitoring, and also analyzing cognitive data at both the group mean and individual classification level. With regard to the seizure onset groups, it was hypothesized that patients with TLE would perform worse on intra-task measures of temporal function (i.e., memory retention including recall and recognition), as well as confrontation naming, while FLE patients would perform worse on intra-task measures that rely more on executive functioning (i.e., immediate attention and learning capacity), as well as verbal fluency (see Supplementary Material). When categorized based on their respective irritative networks, it was anticipated that patients with localized interictal activity (i.e., IEDs limited to the frontal or temporal lobe) would display predominantly frontal or temporal profiles of impairment, respectively, while patients with widespread irritative networks (i.e., IEDs in frontal *and* temporal regions of interest; see Supplementary Material) would demonstrate impairment in both cognitive domains. To further investigate the impact of network dynamics on cognition, patients with frontal and temporal IEDs were subdivided into two groups (temporal seizure onset with frontal propagation or frontal lobe onset with temporal propagation), comparing their performance against normative standards and patients with isolated frontal and temporal interictal activity. It was hypothesized that these subdivided groups would display different cognitive profiles of impairment based on the influence of the ictal focus and wider irritative network activity.

## 2 Methods

### 2.1 Participants

A total of 148 adult patients underwent SEEG monitoring at either Westmead Comprehensive Epilepsy Centre in Sydney, Australia, or the Mater Hospital in Brisbane, Australia, between 2012 and 2023. These patients were investigated for various focal epilepsies, however, only patients with confirmed TLE or FLE (based on the findings from their SEEG evaluations) were included in this study. Patients with SEEG evidence of multifocal seizure onset, seizure onset localized outside the frontal or temporal lobe, or non-localizable seizure onset following SEEG, were excluded. Furthermore, patients were excluded if they had previously undergone epilepsy surgery prior to neuropsychological assessment, had a history of a major psychiatric disorder that required hospitalization, previous severe traumatic brain injury, a history of Psychogenic Non-Epileptic Seizures (PNES) or neurodevelopmental disorder, or an estimated IQ below 70 (based on a measure of estimated premorbid intellect).

Of the 148 patients who underwent SEEG between 2012 and 2023, 51 (34%) met the inclusion criteria. Patients were typically excluded because their seizures were localized to regions outside the frontal or temporal lobes, or SEEG was unable to localize a definite EZ. Three patients were excluded from *seizure onset analyses* as they were found to have multifocal seizure onset (i.e., separate seizure onset zones in frontal and temporal structures), leaving a total of 48 patients. For the *interictal analyses*, an additional four patients were excluded as they did not have an electrode implanted in *both* a frontal and temporal region of interest (see Supplementary Material), leaving a total of 44 patients. This additional exclusion criterion was used to reduce potential sampling bias, as the absence of interictal activity in frontal or temporal regions could not be established if the regions were not implanted.

#### 2.1.1 Seizure onset groups

Of the final seizure onset sample, 15 patients had a frontal lobe seizure onset (FLE), while 33 had temporal lobe seizure onset (TLE). The average number of seizures recorded during the monitoring period for each patient was 17. There were no significant differences in demographic characteristics between the FLE and TLE groups (*p*-values>.170). However, the average age of onset was younger in FLE patients, compared with TLE patients (Mann-Whitney U = 197.5, *z* = 2.07, *p* = .038), and estimated premorbid intelligence was lower in FLE patients, compared with TLE patients (U = 81.5, *z* = 2.91, *p* = .003; see Table 1).

**TABLE 1:** Demographic and clinical characteristics of seizure onset groups.

	TLE (*n* = 33)	FLE (*n* = 15)	*p*-values
Sex Males: *n* (%) Females: *n* (%)	16 (48.5) 17 (51.5)	10 (66.7) 5 (33.3)	.351
Age at neuropsychological assessment: *M (SD)*	33.9 (13.2)	30.1 (9.5)	.477
Years of education: *M (SD)*	12.6 (2.0)	11.5 (1.8)	.090
Age of onset: *M (SD)*	20.7 (14.1)	11.7 (7.9)	**.038**
Epilepsy duration (years): *M (SD)*	12.7 (9.0)	18.1 (9.1)	.065
Febrile seizures in childhood: *n* (%)	4 (12.1)	0 (0.0)	.294
Seizure frequency per year: *M (SD)*	156.9 (174.7)	379.7 (380.9)	.093
Secondary generalised seizures: *n* (%)	13 (39.4)	10 (66.7)	.120
Hippocampal/mesial temporal sclerosis: *n* (%)	6 (18.2)	0 (0.0)	.090
Video-EEG Diagnosis: *n* (%) Frontal lobe Temporal lobe Network (e.g., frontotemporal) Other (e.g., parietal, occipital) Non-localizable	0 (0.0) 10 (33.3) 16 (48.5) 3 (9.1) 4 (12.1)	6 (40.0) 1 (6.7) 4 (26.7) 1 (6.7) 3 (20.0)	N/A
SEEG EZ: *n* (%) Mesial temporal (hipp/ent, amyg) Temporal pole Basal temporal Anterior cingulate Orbitofrontal SMA Lateral PFC	27 (81.8) 2 (6.1) 4 (12.1) - - - -	- - - 8 (53.3) 5 (33.3) 1 (6.7) 1 (6.7)	N/A
SEEG IZ^a^: *n* (%) Temporal only Temporal-frontal (TL-F) Frontal only Frontal-temporal (FL-T)	13 (29.5) 17 (38.6) - -	- - 5 (11.4) 9 (20.4)	N/A
Language representation: *n* (%) Left Right Bilateral	32 (97.0) 0 (0.0) 1 (3.0)	15 (100.0) 0 (0.0) 0 (0.0)	1.000
EZ hemispheric lateralisation: *n* (%) Left hemisphere Right hemisphere Bilateral onset	17 (51.5) 1 (45.5) 1 (3.0)	8 (53.3) 6 (40) 1 (6.7)	.759
Number of current ASMs: *M (SD)*	2.9 (0.9)	3.1 (1.0)	.361
Number of Electrodes: *M (SD)*	13.3 (2.9)	14.8 (2.9)	.094
Days of SEEG monitoring: *M (SD)*	7.2 (3.5)	8.1 (4.5)	.542
Estimated premorbid IQ index (TOPF/NART-R/FSIQ/GAI): *M (SD)*	96.4 (10.1)	87.4 (7.0)	**.003**

Note. TLE, temporal lobe epilepsy; FLE, frontal lobe epilepsy; EZ, epileptogenic zone; IZ, irritative zone; EEG, electroencephalography; SEEG, stereoelectroencephalography; hipp = hippocampus; ent = entorhinal; amyg = amygdala; ASMs, anti-seizure medication; TOPF, test of premorbid functioning; NART-R, National Adult Reading Test-Revised; FSIQ, Full Scale IQ; GAI, general ability index. Bold font = *p* < 0.05.

^a^ Four patients excluded for not having electrodes present in both temporal and frontal regions of interest.

#### 2.1.2 Irritative groups

For the interictal analyses, patients were categorized into groups depending on whether intra-hemispheric irritative activity was isolated to the frontal lobe (FL), the temporal lobe (TL) or present in both the frontal and temporal lobes (FT). The FT group was further subdivided depending on whether they had seizure onset in the temporal lobe with interictal involvement of frontal regions (TL-F), or frontal lobe seizure onset with interictal activity in temporal regions (FL-T). Intra-hemispheric activity was specifically used to categorize groups as studies have found higher incidences of spike and wave complexes ipsilateral to the EZ (Dinkelacker et al., 2016). Furthermore, studies utilizing depth electrodes have found that the most common route of ictal spread in TLE is to the ipsilateral frontal lobe (Lieb et al., 1991; Mayanagi et al., 1996). This same approach to categorising patients has been used in previous studies investigating network activity beyond the temporal lobe (e.g., Barba et al., 2016).

Of the final interictal sample, 5 patients (11%) had epileptogenic activity isolated to the frontal lobe, 13 (30%) had activity isolated to the temporal lobe and 26 (59%) had interictal activity in both the frontal and temporal lobes (i.e., FT). Of the 26 patients in the FT group, 17 (65%) were identified as having temporal lobe onset with propagation to frontal structures (TL-F), while 9 (35%) had frontal lobe onset with temporal propagation (FL-T).

### 2.2 Neuropsychological measures

Standardized neuropsychological testing was undertaken with each patient as part of their presurgical evaluation. See Supplementary Material for a description of each measure included in the current study, as well the standard presurgical evaluation procedure. In summary, all patients completed the Rey Auditory Verbal Learning Test (RAVLT; Rey, 1964), letter fluency (FAS from the Controlled Oral Word Association Test; COWAT; Benton et al., 1994), category fluency (animals) and the Boston Naming Test (BNT; Kaplan et al., 1983). Of note, certain intra-task scores were used from the RAVLT, these being: Trial 1 (immediate attention), Trials 1–5 (sum of correctly recalled words across 5 trials; learning capacity), immediate retention (percentage of Trial 5 items recalled on Trial 6), delayed retention (percentage of Trial 5 items recalled after 30 min on Trial 7) and recognition (sum of true positives after 30 min).

Given the purpose of this study was to differentiate localized dysfunction between groups, neuropsychological measures were categorized based on whether they typically localize (dys) function to the frontal or temporal lobe (McDonald et al., 2001; Saling, 2009; Schraegle et al., 2016; Bremm et al., 2019; Hermann et al., 2020). In summary, measures of frontal function included immediate attention and learning capacity from the RAVLT, letter and category fluency, while temporal measures were immediate retention, delayed retention and recognition from the RAVLT, and naming.

### 2.3 Data analysis

Total test scores from each patient were converted to age-adjusted, and sex-adjusted (where appropriate), z-scores using published normative datasets (Schmidt, 1996; Tombaugh and Hubley, 1997; Tombaugh et al., 1999). These normative datasets were used to establish the population mean (z = 0) for later comparison, as utilized in Knopman et al. (2014). All analyses utilized nonparametric methods and exact tests, due to small samples and non-normally distributed data (according to the Shapiro-Wilk test) and, unless otherwise stated, were performed using STATA 17 (StataCorp, 2021).

Cognitive performances were analyzed relative to normative standards (i.e., z = 0) and also between groups (i.e., statistical comparisons between scores). The purpose of this was to investigate both the cognitive profiles of the groups, as well as distinguishing factors between groups based on cognitive deficits. Both strategies have been used widely within the literature when attempting to discern the localizing value of cognitive measures.

For continuous data, Wilcoxon signed-rank tests were used to investigate the profiles of each group compared to the normative sample mean (z = 0). Wilcoxon rank-sum tests (via the Mann-Whitney U statistic) were used to compare the z-scores between two groups (e.g., FLE vs. TLE) and Kruskal-Wallis tests were used when comparing z-scores for more than two groups (e.g., FL vs. TL vs. FT). The effect sizes were interpreted according to the benchmarks outlined by Cohen (1988).

Cognitive variable z-scores were also categorized into level of impairment: no cognitive impairment (z-score above −1), mild impairment (z-score of −1 to −1.63), or moderate to severe impairment (z-score of −1.64 or below). Each patient was categorized based on their individual performance, rather than the group mean. This methodology was also used by Knopman et al. (2014) and is common in clinical practice. Simultaneous 95% confidence intervals for multinomial proportions were calculated for the proportion of patients in each cognitive impairment group using the Wilson score interval method (Wilson, 1927) via the R DescTools package (R Core Team, 2021; Signorell et al., 2021). This method has the advantage of correcting for multiple tests while performing well with small sample sizes (Dean and Pagano, 2015). These intervals were compared with the expected proportion of mild and moderate/severe impairment in the normal population (i.e., 84% no impairment, 11% mild impairment, 5% moderate/severe impairment). Intervals excluding the population proportion were deemed significant.

When comparing cognitive performance to normative standards (e.g., z = 0), cognitive deficits were only interpreted where significant differences were observed on *both* continuous and categorical analyses. This approach was employed to increase the robustness of the findings, reduce the chance of overinterpreting impairment relative to the normative sample (particularly considering the small group sizes), and to control for the effects of multiple comparisons.

## 3 Results

### 3.1 Cognition based on seizure onset alone

**FIGURE 3:**
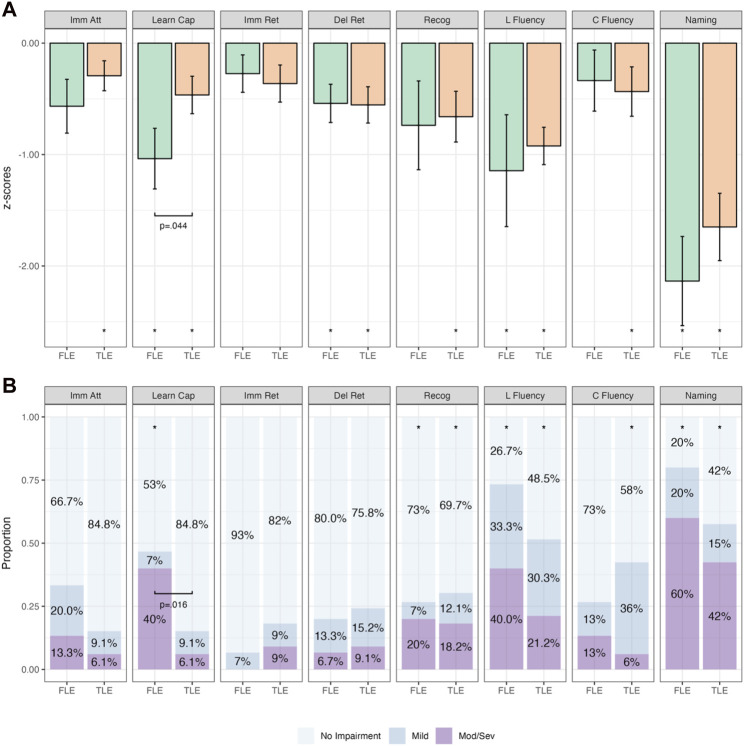
Comparative Continuous (Group z-score Means; **(A)** and Categorical (Proportions; **(B)**) Cognitive Outcomes in Temporal Lobe Epilepsy (TLE; *n* = 33) and Frontal Lobe Epilepsy (FLE; *n* = 15) Patients. *Note.* Bars in **(A)** represent z-score means with error bars showing one standard error. Imm Att, immediate attention (Trial 1); Learn Cap, learning capacity (Trials 1–5); Imm Ret, immediate retention; Del Ret, delayed retention; Recog, recognition; L Fluency, letter fluency; C Fluency, category fluency. *p < .05 relative to population (z = 0). Brackets with *p-*values represent statistically significant differences between groups (*p* < .05).

Groups were first analyzed when categorized purely on the basis of seizure onset (i.e., not taking into account the irritative network). The findings from group mean and categorical analyses within and between FLE and TLE groups are presented in Figure 3 (and Supplementary Tables S1–S3). FLE and TLE groups performed significantly worse than the normative sample on several cognitive variables, across both continuous and categorical analyses (Supplementary Table S1). On continuous variable analyses, FLE patients performed significantly worse than the normative sample on learning capacity (RAVLT Trials 1–5; *p* = .002), delayed retention (*p* = .006), letter fluency (*p* = .015), and naming (*p* < .001). TLE patients performed significantly worse on all cognitive variables (*p*-values ≤ .035), except for immediate recall (*p* = .073). On categorical analyses, the FLE group demonstrated a significantly higher percentage of mild impairment (than the expected 11%) on letter fluency (15%–58%), and a higher percentage of moderate/severe impairment (expected 5%) on learning capacity (20%–64%), recognition (7%–45%), letter fluency (20%–64%), and naming (36%–80%). TLE patients demonstrated a significantly higher percentage of mild impairment on letter fluency (17%–47%) and category fluency (22%–53%), and a higher percentage of moderate/severe impairment on recognition (9%–34%), letter fluency (11%–38%), and naming (27%–59%).

When combining the findings of both continuous and categorical analyses, the FLE group performed statistically significantly worse on measures of learning capacity, letter fluency, and naming, while the TLE group performed statistically significantly worse on measures of recognition, letter fluency, category fluency, and naming.

When comparing the mean z-scores between FLE and TLE patients (Supplementary Table S2), only learning capacity was significantly different between groups, with the FLE group performing worse than the TLE group (*p* = .044). This finding was also observed on categorical analyses (Supplementary Table S3), with Fisher’s exact test indicating a higher proportion of patients with more severe impairment on learning capacity in the FLE group compared with the TLE group (*p* = .016).

### 3.2 Cognition based on irritative network

Analysis was then performed when the patients were separated into groups based on the wider epileptic network/irritative network. Different profiles of cognitive impairment were found for each of the initial irritative groups (i.e., FL, TL, and FT) compared with normative standards (z = 0; see Supplementary Table S4). While the proportion of cases with moderate/severe impairment on letter fluency (ranging from 12% to 77%) and naming (12%–77%) was significantly higher in the FL group compared with normative expectations, there was no evidence that the mean z-score differed from the population on continuous analyses (*p*-values>.062). TL patients performed significantly worse than the population on naming across both sets of analyses, while the FT group performed significantly worse on recognition, letter, category fluency and naming. The FT group also performed significantly worse on immediate attention, learning capacity, and immediate and delayed recall, on group mean analyses (*p*-values ≤ .003), however the proportion of impaired performance was not statistically significant on categorical analyses. The findings indicate that the TL group was specifically impaired on naming, while the FT group was impaired on both temporal and frontal measures across continuous and categorical analyses. When these groups were compared to one another (Supplementary Tables S5, S6), the FT group performed worse on immediate retention (*p* = .048) and recognition (*p* = .038), compared with both FL and TL groups.

**FIGURE 4:**
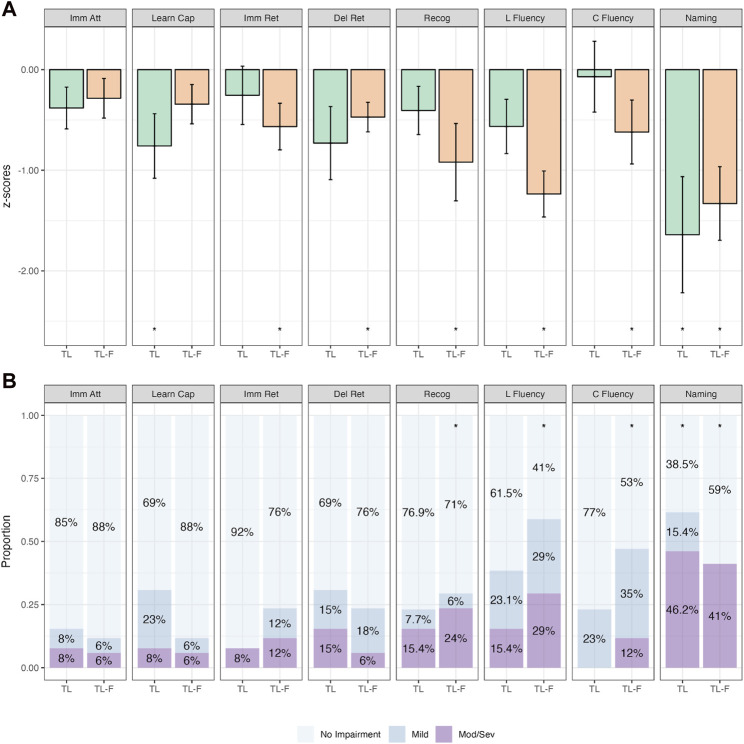
Comparative Continuous (Group z-score Means; **(A)**) and Categorical (Proportions; **(B)**) Cognitive Outcomes in Patients with Isolated Temporal Irritative Networks (TL; *n* = 26) and Temporal to Frontal Irritative Networks (TL-F; *n* = 17). Note. Bars in **(A)** represent z-score means with error bars showing one standard error. Imm Att, immediate attention (Trial 1); Learn Cap, learning capacity (Trials 1–5); Imm Ret, immediate retention; Del Ret, delayed retention; Recog, recognition; L Fluency, letter fluency; C Fluency, category fluency. *p<.05 relative to population (z=0).

The next phase of analyses was undertaken with the FT group subdivided based on the propagation pattern (i.e., TL-F & FL-T). Figure 4 depicts the mean z-scores for TL and TL-F groups. The TL-F group performed significantly worse on recognition, letter fluency, category fluency and naming across both continuous and categorical analyses (*p*-values ≤.017; Supplementary Table S7). There were no statistically significant differences when comparing TL and TL-F groups directly (*p*-values>.110; Supplementary Tables S8 & S9).

**FIGURE 5:**
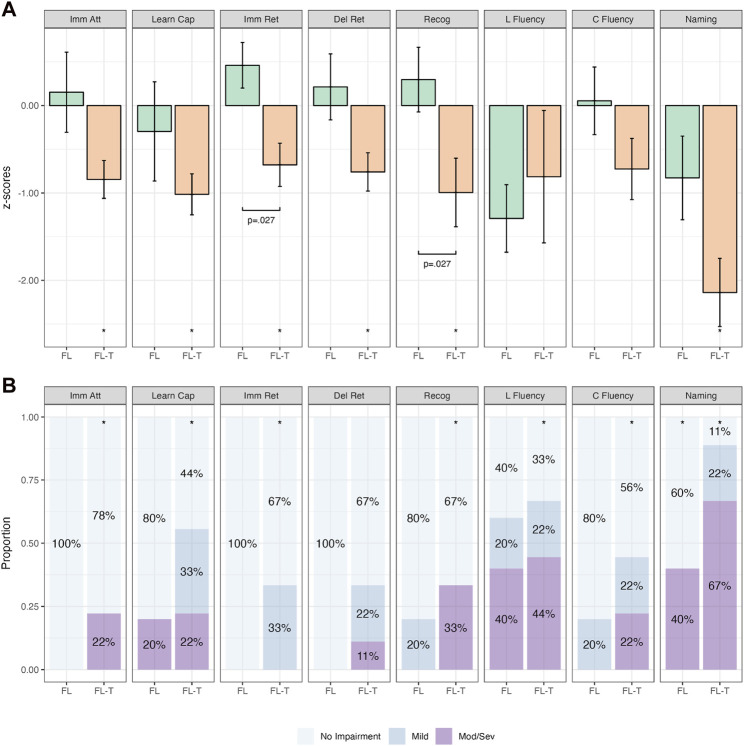
Comparative Continuous (Group z-score Means; **(A)**) and Categorical (Proportions; **(B)**) Cognitive Outcomes in Patients with Isolated Frontal Irritative Networks (FL; *n* = 5) and Frontal to Temporal Irritative Networks (FL-T; *n* = 9). Note. Bars in **(A)** represent z-score means with error bars showing one standard error. Imm Att, immediate attention (Trial 1); Learn Cap, learning capacity (Trials 1-5); Imm Ret, immediate retention; Del Ret, delayed retention; Recog, recognition; L Fluency, letter fluency; C Fluency, category fluency. *p < .05 relative to population (z=0). Brackets with *p*-values represent statistically significant differences between groups (*p* < .05).

Figure 5 depicts the performance between the FL and FL-T groups. Patients in the FL-T group performed significantly worse on immediate attention, learning capacity, immediate retention, recognition and naming across both continuous and categorical analyses (*p*-values ≤.039; Supplementary Table S7). When compared directly, the FL-T group performed significantly worse on immediate retention and recognition on group mean analyses (*p*-values = .027), with delayed retention and naming approaching significance (*p*-values = .059; Supplementary Tables S10, S11).

**FIGURE 6:**
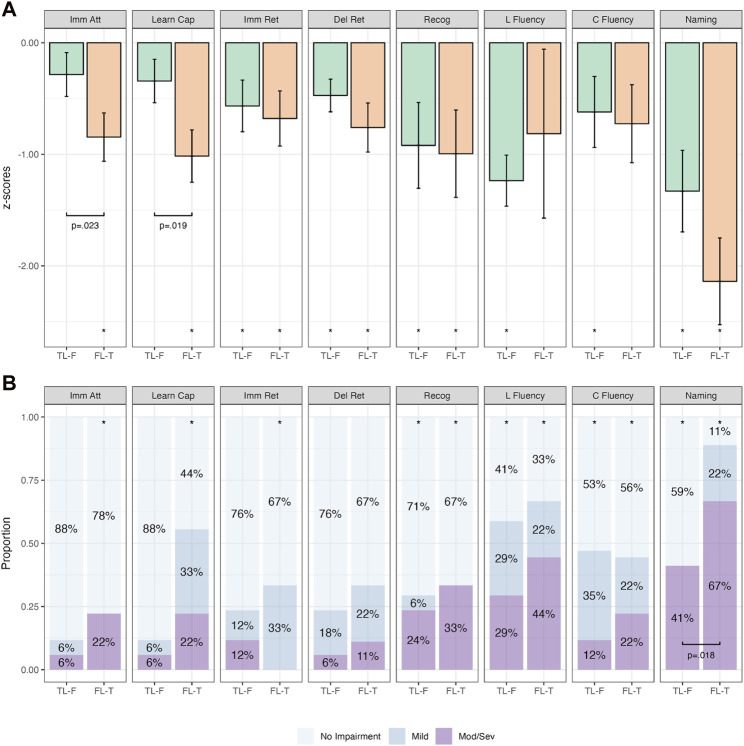
Comparative Continuous (Group z-score Means; **(A)**) and Categorical (Proportions; **(B)**) Cognitive Outcomes in subdivided frontotemporal groups. Note. Bars in **(A)** represent z-score means with error bars showing one standard error. Imm Att, immediate attention (Trial 1); Learn Cap, learning capacity (Trials 1–5); Imm Ret, immediate retention; Del Ret, delayed retention; Recog, recognition; L Fluency, letter fluency; C Fluency, category fluency. *p < .05 relative to population (z = 0). Brackets with *p*-values represent statistically significant differences between groups (*p* < .05).

Figure 6 depicts the cognitive performance between the TL-F and FL-T groups. There was a significant difference between these groups on immediate attention (*p* = .023) and learning capacity (*p* = .019), whereby the FL-T group performed worse across both on group mean analyses (Supplementary Table S12). On categorical analyses, the proportion of impairment on naming was significantly higher in the FL-T group compared to the TL-F group (*p* = .018). Learning capacity also trended in the same direction, however failed to reach statistical significance (*p* = .056; Supplementary Table S13).

## 4 Discussion

The present study aimed to investigate cognitive impairment in SEEG patients when categorized based on the seizure onset zone *or* the irritative network. Speaking to the former, while subtle differences were observed in the cognitive profiles of FLE and TLE patients, the only significant difference *between* the groups was that patients with FLE had reduced learning capacity (i.e., there was no significant difference across all other domains). When patients were categorized based on the distribution of their epileptic network (i.e., irritative network), more circumscribed profiles of impairment were observed in patients with networks limited to either frontal or temporal structures, while those with broad involvement of the frontotemporal network (i.e., FT) performed significantly worse across a number of cognitive domains, including measures of executive functioning, memory, and language. Further subdivision of the FT group revealed the impact of activity beyond the site of seizure onset, as patients with frontal seizure onset were more likely to display memory impairment where the irritative network involved temporal structures (i.e., FL-T vs FL), and patients with temporal seizure onset were more likely to demonstrate executive dysfunction where there was irritative extension to the frontal lobe (i.e., TL-F vs TL).

The following study extended on previous research by investigating cognitive impairment relative to normative expectations at both the group mean (continuous) and individual classification level (categorical). Table 2 summarizes the cognitive measures that were impaired across *both* continuous and categorical analyses for each group. Comparing the findings between groups, several putative measures of temporal and frontal function revealed wider network activity (i.e., irritative activity), as opposed to localized dysfunction (i.e., seizure onset). In terms of temporal measures, recognition memory for a word list was initially found to be significantly impaired in the TLE group, however, impairment on this measure was only observed in the TL-F and FL-T groups (i.e., those with frontotemporal network involvement). Similarly, while naming was impaired in both TLE and FLE groups, it was subsequently only observed in the TL, TL-F and FL-T groups. These findings support recent phenotype studies that have demonstrated naming impairment in FLE patients (Arrotta et al., 2022), and further suggest that this may be due to the involvement of temporal structures within the wider seizure network (i.e., the FL-T group).

**TABLE 2:** Table of statistically significant cognitive impairment on both continuous and categorical analyses when compared to the normative sample.

	Seizure onset groups		Irritative groups			Subdivided FT groups	
Cognitive Domain	FLE	TLE	FL	TL	FT	TL-F	FL-T
Immediate Attention							X
Learning Capacity	X						X
Immediate Recall							X
Delayed Recall							
Recognition		X			X	X	X
Letter Fluency	X	X			X	X	
Category Fluency		X			X	X	
Naming	X	X		X	X	X	X

*Note*. X = statistically significant across both continuous and categorical analyses. FLE, frontal lobe epilepsy; TLE, temporal lobe epilepsy; FL, isolated frontal lobe irritative network; TL, isolated temporal irritative network; FT, frontotemporal irritative network; TL-F, temporal to frontal irritative network; FL-T, frontal to temporal irritative network.

Several frontal measures also provided insights into network dysfunction. For example, while letter fluency was impaired in TLE patients, subsequent analyses revealed it was only impaired in the TL-F group (i.e., where there was network propagation to frontal regions). This same pattern was observed in patients with impaired category fluency, providing support for the growing body of literature demonstrating frontotemporal network involvement during verbal fluency tasks (Wang et al., 2010; Whiteside et al., 2015). Finally, while learning capacity was specifically impaired in FLE patients, the findings from the irritative analyses suggest that this was only evident in the FL-T group. Taken together, these findings suggest that both the regional involvement and directionality of the irritative network influence the profile of cognitive impairment, and potentially shed light on recent studies that have found similar profiles of cognitive impairment in patients with focal and generalized epilepsies (Gauffin et al., 2022).

The use of an exclusive SEEG sample was central to the current study. While previous studies have investigated the impact of interictal activity on cognition, most of these studies have identified IEDs using scalp-EEG, which has been found to underestimate their presence, particularly when emanating from mesial structures, such as those commonly involved in TLE and FLE (Merlet et al., 1998; Spencer et al., 1998; Pyrzowski et al., 2021). The breakdown of the irritative groups within the following study is worthy of brief discussion. The proportions of patients with isolated frontal, temporal or frontotemporal interictal activity within the current sample were 11%, 30%, and 59%, respectively. These proportions are considerably different from previous research exploring the influence of interictal activity on cognition using scalp-EEG. For example, in a recent study investigating the impact of IEDs on cognition in children, only 12% of the sample were identified as having frontotemporal interictal activity (Glennon et al., 2016). In adults, a study by Vlooswijk et al. (2011) found a more balanced representation of temporal (28%), frontal (36%) and frontotemporal groups (36%), when examining working memory performance in patients with focal epilepsy. The current sample included a much larger proportion of patients with frontotemporal IEDs, which may be due to the added localizing value of SEEG, as depth electrodes are able to sample anatomically deep structures that are otherwise undetectable on scalp-EEG (e.g., orbitofrontal cortex, anterior cingulate; Suresh et al., 2015). This supports the notion that previous research which has identified IEDs using scalp-EEG may have underestimated the true involvement of frontal and temporal structures, and subsequently the potential contribution to cognitive impairment where patients have been categorized according to seizure onset alone.

While the manifestation of cognitive impairment in epilepsy is largely associated with the location of seizure activity, certain factors, such as age of onset, duration of epilepsy, and frequency of seizures, have been found to impact the severity of cognitive deficits (Holmes, 2015; DeGeorge et al., 2021; Novak et al., 2022). Patients in the FLE group did have an earlier age of onset and lower estimated premorbid intelligence compared with the TLE group. While not statistically significant, there was also a trend for patients in the FLE group to have a higher seizure frequency. Previous research has found poorer performance on measures of general intellect in patients with FLE (Braakman et al., 2012), with some studies attributing this to a typically younger age of seizure onset (Campiglia et al., 2014). Indeed, previous studies have indicated that earlier onset of seizures is associated with a greater impact on cognition (particularly working memory and executive function; Black et al., 2010). As such, the difference in age of onset between the TLE and FLE groups may have provided some contribution to the overall findings. Alternatively, the association between age of seizure onset and estimated premorbid intellect may be due to the impact of early seizure onset (≈11 years the FLE group) on vocabulary acquisition—potentially resulting in an underestimation of premorbid IQ in the current sample, as intellect was primarily estimated using measures of vocabulary (i.e., the TOPF and NART-R). Unfortunately, given the size of the overall sample, investigation of potentially moderating clinical factors between epilepsy types was limited.

### 4.1 Clinical implications

The findings from the current study highlight the importance of the epileptic network/irritative zone in the determination of cognitive impairment for patients with medically-refractory epilepsy. This concept is an evolution of the structure-function interpretation that has been traditionally used to localize seizures in clinical settings. Spencer (2002) postulated that seizures can occur from different nodes within the same epileptic network, including regions beyond the focal site of onset, and remain clinically indistinguishable from one another as the whole network is responsible for the manifestation of the seizure. As such, accurately identifying the areas involved in the wider network has significant implications for surgical outcomes.

Temporal plus epilepsy (TPE) represents an excellent clinical example for the importance of establishing the wider boundaries of an individual’s seizure network prior to epilepsy surgery. TPE refers to a focal epilepsy where the primary EZ extends beyond the temporal lobe to other neighboring extratemporal regions (including perisylvian and orbitofrontal cortices; Barba et al., 2007; Ryvlin and Kahane, 2005). The concept of TPE was established in response to recurrent failed temporal lobectomies in patients with wider seizure networks (Barba et al., 2016). Failure to identify TPE carries significant consequences for surgical success, with some studies observing only 9% of TPE patients achieving an Engel Class Ia outcome (completely seizure free; no auras) following standard anterior lobectomy, compared with 74% of patients whose surgery was tailored to the wider seizure network (Ryvlin et al., 2001). The diagnosis of TPE is further complicated by the fact that patients are commonly MRI-negative and have scalp-EEG features almost indistinguishable from classic TLE (Barba et al., 2007). As such, TPE can only be confirmed through SEEG, as invasive recording allows for simultaneous sampling of the temporal lobe and neighboring areas of cortex (e.g., frontal, parietal, and occipital cortices; Andrade-Machado and Benjumea-Cuartas, 2016).

Advances in structural and functional neuroimaging have reduced the reliance on neuropsychological assessment to localize the EZ (Baxendale, 2018). Indeed, surgical outcomes are superior where a causative lesion has been established on neuroimaging (McIntosh et al., 2001; Téllez-Zenteno et al., 2010; Oldan et al., 2018). While some patients may progress directly to surgery following an initial presurgical evaluation (i.e., where there is concordance among presurgical non-invasive investigations), others will require additional invasive investigations. In this scenario, successful surgical outcomes hinge on the accuracy of the electrode implantation, which aims to capture an individual’s entire seizure network, to accurately discern the boundaries of their EZ (Bonini et al., 2014; Gonzalez-Martinez, 2016; Chauvel, 2020). Where an epileptogenic lesion has not been established, the onus is placed on the remaining non-invasive investigations (i.e., neuropsychological assessment, clinical semiology) to develop the implantation plan. The findings from the current study suggest that neuropsychological assessment provides insight into regions involved in an individual’s wider seizure network. As such, an individual’s pre-implantation cognitive profile may assist with identification of regional involvement beyond the site of suspected seizure onset (e.g., TPE). For example, a more broad implantation may be required where a patient with suspected TLE displays executive dysfunction and frontal seizure semiology.

### 4.2 The use of continuous and categorical data

The use of continuous and categorical data was an important feature of the current study and novel in the context of previous cognitive research—which typically uses one approach to interpret impairment. Our findings demonstrate how the interpretation of continuous and categorical data can produce different profiles of impairment compared with normative standards (z = 0). The decision to analyze both sets of data was based on their various advantages and disadvantages. Continuous analyses, which are commonly used within cognitive research, allow for the consideration of potentially important variability within the data. However, this use of group mean data has been criticized for hampering diagnostic precision, as it dilutes the contribution of each individual’s data (Reyes et al., 2020; Caciagli and Bassett, 2022; Lee et al., 2022). In contrast, consideration of categorical data is more akin to clinical interpretation of cognitive impairment, as the individual’s categorisation is compared to normative expectations. Criticisms of this approach include a lack of flexibility, due to the categorization of function as either impaired or intact. There is also ongoing debate regarding the ideal cut-off score for impairment, with the majority of studies utilizing 1 or 1.5 SDs below the mean (e.g., Suresh et al., 2015; Bremm et al., 2019; Reyes et al., 2020). Unfortunately, very little has been published on the differences between these approaches in the context of interpreting cognitive impairment.

Given the small sample size of the current study, corrections for multiple comparisons were not made, as they would have likely minimized any chance of observing trends within the data. To mitigate against this omission, cognitive impairment relative to normative expectations was only inferred where deficits were observed on both continuous and categorical analyses, as a means to increase the robustness of the findings. The utility of this approach should be investigated in future research.

### 4.3 Limitations

There are some limitations to the following study that should be addressed. First, the sample size was small given SEEG is an emerging procedure in Australia. As such, the numbers in each group were not balanced. This was particularly evident in the frontal groups (i.e., FLE, FL, and FL-T) and probably impacted our ability to observe differences between groups due to reduced statistical power. The small sample sizes also impacted our ability to explore further categorization of groups based on seizure laterality (e.g., right FLE, left FLE, etc.), handedness/dominance, involvement of unilateral or bilateral structures, and sub-lobar involvement (e.g., orbitofrontal vs. dorsolateral PFC in frontal lobe groups). While the current study focused on the involvement of frontal and temporal regions, future studies may want to investigate the network involvement beyond these areas (i.e., extending posteriorly to parietal and occipital regions). More distal network involvement may result in different profiles and/or more severe cognitive deficits. Despite these limitations, the study remains the first of its kind to explore neuropsychological assessment exclusively in SEEG patients, to the authors’ knowledge.

The multi-center nature of this study was a strength but introduced both neuropsychological and SEEG variability. The preference of executive measures differed between sites, limiting our ability to utilize a large battery of executive tasks. The standardized inclusion of additional measures of executive functioning may have resulted in the emergence of more defined profiles of impairment. Similarly, the SEEG implantation strategies were patient specific and not standardized between centers. Sampling was more than sufficient to determine if a lobe was a part of the irritative network, however more detailed determination of the volume of involvement, which specific structures within that lobe were involved, and/or quantifying the degree of involvement were not possible. If this study were repeated, utilizing a large dataset with a standardized approach to neuropsychological assessment and SEEG implantation would be highly beneficial.

## 5 Conclusion

Patients with SEEG evidence of a widespread epileptic network had more widespread and severe cognitive impairment than those with seizures limited to either frontal or temporal regions. Furthermore, our findings suggest that the directionality of propagating network activity appears to influence the profile of cognitive impairment, even where the same regions are involved (i.e., frontotemporal network activity). While cognitive impairment has traditionally been used to establish the node of seizure onset, our findings suggest that it may provide more useful insight into the wider boundaries and features of the seizure network. Understanding this pattern of cognitive deficits may assist with identifying patients with a more diffuse epilepsy network where greater SEEG sampling may assist with improving outcomes.

## Data Availability

The raw data supporting the conclusions of this article will be made available by the authors, without undue reservation.

## References

[B1] Agah E. Asgari-Rad N. Ahmadi M. Tafakhori A. Aghamollaii V. (2017). Evaluating executive function in patients with temporal lobe epilepsy using the frontal assessment battery. Epilepsy Res. 133, 22–27. 10.1016/j.eplepsyres.2017.03.0112017.03.011 28407518

[B2] Andrade–Machado R. Benjumea-Cuartas V. (2016). Temporal plus epilepsy: anatomo-electroclinical subtypes. Iran. J. Neuro. 15 (3), 153–163. Available at: https://pubmed.ncbi.nlm.nih.gov/27648177. PMC5027151 27648177

[B3] Arrotta K. Reyes A. Kaestner E. McDonald C. R. Hermann B. P. Barr W. (2022). Cognitive phenotypes in frontal lobe epilepsy. Epilepsia 63 (7), 1671–1681. 10.1111/epi.17260 35429174 PMC9545860

[B4] Barba C. Barbati G. Minotti L. Hoffmann D. Kahane P. (2007). Ictal clinical and scalp-EEG findings differentiating temporal lobe epilepsies from temporal “plus” epilepsies. Brain 130 (7), 1957–1967. 10.1093/brain/awm108 17535836

[B5] Barba C. Rheims S. Minotti L. Guénot M. Hoffmann D. Chabardès S. (2016). Temporal plus epilepsy is a major determinant of temporal lobe surgery failures. Brain 139 (2), 444–451. 10.1093/brain/awv372 26700686

[B6] Baxendale S. (2018). Neuropsychological assessment in epilepsy. Pract. Neurol. 18 (1), 43–48. 10.1136/practneurol-2017-001827 29326240

[B7] Benton A. L. Hamsher K. Rey G. L. Sivan A. B. (1994). Multilingual aphasia examination. 3rd Edn. Iowa City, IA: AJA Associates.

[B8] Besson P. Dinkelacker V. Valabregue R. Thivard L. Leclerc X. Baulac M. (2014). Structural connectivity differences in left and right temporal lobe epilepsy. NeuroImage 100, 135–144. 10.1016/j.neuroimage.2014.04.071 24814212

[B9] Bettus G. Ranjeva J. P. Wendling F. Bénar C. G. Confort-Gouny S. Régis J. (2011). Interictal functional connectivity of human epileptic networks assessed by intracerebral EEG and BOLD signal fluctuations. PLoS One 6 (5), e20071. 10.1371/journal.pone.0020071 21625517 PMC3098283

[B10] Black L. C. Schefft B. K. Howe S. R. Szaflarski J. P. Yeh H. S. Privitera M. D. (2010). The effect of seizures on working memory and executive functioning performance. Epilepsy & Behav. 17 (3), 412–419. 10.1016/j.yebeh.2010.01.006 20153981

[B11] Bonini F. McGonigal A. Trébuchon A. Gavaret M. Bartolomei F. Giusiano B. (2013). Frontal lobe seizures: from clinical semiology to localization. Epilepsia 55 (2), 264–277. 10.1111/epi.12490 24372328

[B12] Bou Assi E. Zerouali Y. Robert M. Lesage F. Pouliot P. Nguyen D. K. (2020). Large-scale desynchronization during interictal epileptic discharges recorded with intracranial EEG. Front. Neurol. 11, 529460. 10.3389/fneur.2020.529460 33424733 PMC7785800

[B13] Braakman H. M. Ijff D. M. Vaessen M. J. Debeij-van Hall M. H. Hofman P. A. Backes W. H. (2012). Cognitive and behavioural findings in children with frontal lobe epilepsy. Eur. J. Paediatr. Neurol. 16 (6), 707–715. 10.1016/j.ejpn.2012.05.003 22748634

[B14] Bremm F. J. Hendriks M. P. Bien C. G. Grewe P. (2019). Pre- and postoperative verbal memory and executive functioning in frontal versus temporal lobe epilepsy. Epil. & Behav. 101, 106538. 10.1016/j.yebeh.2019.106538 31678807

[B15] Caciagli L. Bassett D. S. (2022). Epilepsy imaging meets machine learning: a new era of individualized patient care. Brain 145 (3), 807–810. 10.1093/brain/awac027 35307732

[B16] Campiglia M. Seegmuller C. le Gall D. Fournet N. Roulin J. L. Roy A. (2014). Assessment of everyday executive functioning in children with frontal or temporal epilepsies. Epilepsy & Behav. 39, 12–20. 10.1016/j.yebeh.2014.07.023 25150755

[B17] Centeno M. Thompson P. Koepp M. Helmstaedter C. Duncan J. (2010). Memory in frontal lobe epilepsy. Epilepsy Res. 91 (2–3), 123–132. 10.1016/j.eplepsyres.2010.07.017 20800996

[B18] Chauvel P. (2020). The history and principles of stereo EEG. Berlin: Springer eBooks. 10.1891/9780826136930.0001

[B19] Cohen J. (1988). Statistical power analysis for the behavioral sciences. 2nd Edn. London: Routledge.

[B20] Dean N. Pagano M. (2015). Evaluating confidence interval methods for binomial proportions in clustered surveys. J. Surv. Statistics Methodol. 3 (4), 484–503. 10.1093/jssam/smv024

[B21] DeGeorge E. G. Fullen C. Gess J. L. Kleiner J. S. Larson-Prior L. (2021). Effects of age of onset and medication on cognitive performance and quality of life in patients with epilepsy. Epilepsy & Behav. 121, 108008. 10.1016/j.yebeh.2021.108008 34004525

[B22] Dinkelacker V. Xin X. Baulac M. Samson S. Dupont S. (2016). Interictal epileptic discharge correlates with global and frontal cognitive dysfunction in temporal lobe epilepsy. Epilepsy & Behav. 62, 197–203. 10.1016/j.yebeh.2016.07.009 27494355

[B23] Elwan S. So N. K. Enatsu R. Bingaman W. (2013). Pseudotemporal ictal patterns compared with mesial and neocortical temporal ICTal patterns. J. Clin. Neurophys. 30 (3), 238–246. 10.1097/wnp.0b013e3182872f70 23733087

[B24] Fahoum F. Lopes R. Pittau F. Dubeau F. Gotman J. (2012). Widespread epileptic networks in focal epilepsies: EEG-fMRI study. Epilepsia 53 (9), 1618–1627. 10.1111/j.1528-1167.2012.03533.x 22691174 PMC4492710

[B25] Gauffin H. Landtblom A. M. Vigren P. Frick A. Engström M. McAllister A. (2022). Similar profile and magnitude of cognitive impairments in focal and generalized epilepsy: a pilot study. Front. Neurol. 12, 746381. 10.3389/fneur.2021.746381 35095714 PMC8790571

[B26] Glennon J. M. Weiss-Croft L. Harrison S. Cross J. H. Boyd S. G. Baldeweg T. (2016). Interictal epileptiform discharges have an independent association with cognitive impairment in children with lesional epilepsy. Epilepsia 57 (9), 1436–1442. 10.1111/epi.13479 27503785

[B27] González-Martínez J. Bulacio J. Thompson S. E. Gale J. T. Smithason S. Najm I. (2016). Technique, results, and complications related to robot-assisted stereoelectroencephalography. Neurosurgery 78 (2), 169–180. 10.1227/neu.0000000000001034 26418870

[B28] Henin S. Shankar A. Borges H. Flinker A. Doyle W. Friedman D. (2021). Spatiotemporal dynamics between interictal epileptiform discharges and ripples during associative memory processing. Brain 144 (5), 1590–1602. 10.1093/brain/awab044 33889945 PMC8219352

[B29] Hermann B. Conant L. L. Cook C. J. Hwang G. Garcia-Ramos C. Dabbs K. (2020). Network, clinical and sociodemographic features of cognitive phenotypes in temporal lobe epilepsy. NeuroImage Clin. 27, 102341. 10.1016/j.nicl.2020.102341 32707534 PMC7381697

[B30] Holmes G. L. (2015). Cognitive impairment in epilepsy: the role of network abnormalities. Epileptic Disord. 17 (2), 101–116. 10.1684/epd.2015.0739 25905906 PMC5410366

[B31] Jehi L. (2018). The epileptogenic zone: concept and definition. Epilepsy Curr. 18 (1), 12–16. 10.5698/1535-7597.18.1.12 29844752 PMC5963498

[B32] Kaplan E. Goodglass H. Weintraub S. (1983). The Boston Naming Test. Philadelphia, PA: Lea & Febiger.

[B33] Knopman A. A. Wong C. Stevenson R. J. Homewood J. Mohamed A. Somerville E. (2014). The cognitive profile of occipital lobe epilepsy and the selective association of left temporal lobe hypometabolism with verbal memory impairment. Epilepsia 55 (8), e80–e84. 10.1111/epi.12623 24725141

[B34] Lee H. M. Fadaie F. Gill R. Caldairou B. Sziklas V. Crane J. (2022). Decomposing MRI phenotypic heterogeneity in epilepsy: a step towards personalized classification. Brain 145 (3), 897–908. 10.1093/brain/awab425 34849619 PMC9050524

[B35] Lieb J. P. Dasheiff R. M. Engel J. Jr (1991). Role of the frontal lobes in the propagation of mesial temporal lobe seizures. Epilepsia 32 (6), 822–837. 10.1111/j.1528-1157.1991.tb05539.x 1743154

[B36] Lüders H. O. Engel J. Jr Munari C. (1993). “General principles,” in Surgical treatment of the epilepsies. Editor Engel J. Jr (New York, NY: Raven Press), 137–153.

[B37] Lüders H. O. Najm I. Nair D. Widdess-Walsh P. Bingman W. (2006). The epileptogenic zone: general principles. Epileptic Disord. 8 (S1-9), S1–S9. 10.1684/j.1950-6945.2006.tb00204.x 17012067

[B38] Maccotta L. He B. J. Snyder A. Z. Eisenman L. N. Benzinger T. L. Ances B. M. (2013). Impaired and facilitated functional networks in temporal lobe epilepsy. NeuroImage Clin. 2, 862–872. 10.1016/j.nicl.2013.06.011 24073391 PMC3777845

[B39] Mayanagi Y. Watanabe E. Kaneko Y. (1996). Mesial temporal lobe epilepsy: clinical features and seizure mechanism. Epilepsia 37 (Suppl. 3), 57–60. 10.1111/j.1528-1157.1996.tb01823.x 8681916

[B40] McDonald C. R. Bauer R. M. Grande L. Gilmore R. L. Roper S. N. (2001). The role of the frontal lobes in memory: evidence from unilateral frontal resections for relief of intractable epilepsy. Arch. Clin. Neuropsy. 16 (6), 571–585. 10.1093/arclin/16.6.571 14590155

[B41] McIntosh A. M. Wilson S. J. Berkovic S. F. (2001). Seizure outcome after temporal lobectomy: current research practice and findings. Epilepsia 42 (10), 1288–1307. 10.1046/j.1528-1157.2001.02001.x 11737164

[B42] Merlet I. Garcia-Larrea L. Ryvlin P. Isnard J. Sindou M. Mauguière F. (1998). Topographical reliability of mesio-temporal sources of interictal spikes in temporal lobe epilepsy. Electroencephalogr. Clin. Neurophys. 107 (3), 206–212. 10.1016/s0013-4694(98)00055-8 9803951

[B43] Novak A. Vizjak K. Rakuša M. (2022). Cognitive impairment in people with epilepsy. J. Clin. Med. 11 (1), 267. 10.3390/jcm11010267 35012007 PMC8746065

[B44] Oldan J. D. Shin H. W. Khandani A. H. Zamora C. Benefield T. Jewells V. (2018). Subsequent experience in hybrid PET-MRI for evaluation of refractory focal onset epilepsy. Seizure 61, 128–134. 10.1016/j.seizure.2018.07.022 30138825

[B46] Penfield W. (1939). The Epilepsies: with a note on radical therapy. N. Engl. J. Med. 221 (6), 209–218. 10.1056/nejm193908102210601

[B47] Penfield W. Milner B. (1958). Memory deficit produced by bilateral lesions in the hippocampal zone. Arch. Neurol. Psych. 79, 475–497. 10.1001/archneurpsyc.1958.02340050003001 13519951

[B48] Pyrzowski J. Douget J. E. L. Fouad A. Siemiński M. Jędrzejczak J. Van Quyen M. L. (2021). Zero-crossing patterns reveal subtle epileptiform discharges in the scalp EEG. Sci. Rep. 11 (1), 4128. 10.1038/s41598-021-83337-3 33602954 PMC7892826

[B49] Rayner G. Tailby C. (2017). Current concepts of memory disorder in epilepsy: edging towards a network account. Curr. Neurol. Neurosci. Rep. 17 (8), 55. 10.1007/s11910-017-0765-7 28631193

[B50] R Core Team (2021). R: A Language and Environment for Statistical Computing. Vienna, Austria: R Foundation for Statistical Computing. Available at: https://www.R-project.org/.

[B51] Ren Y. Pan L. Du X. Hou Y. Li X. Song Y. (2020). Functional brain network mechanism of executive control dysfunction in temporal lobe epilepsy. BMC Neurol. 20 (1), 137. 10.1186/s12883-020-01711-6 32295523 PMC7161158

[B52] Rey A. (1964). L’examen Clinique Enpsychologie. Paris: Presses Universitaires de France.

[B53] Reyes A. Kaestner E. Ferguson L. Jones J. E. Seidenberg M. Barr W. B. (2020). Cognitive phenotypes in temporal lobe epilepsy utilizing data- and clinically driven approaches: moving toward a new taxonomy. Epilepsia 61 (6), 1211–1220. 10.1111/epi.16528 32363598 PMC7341371

[B54] Ryvlin P. Kahane P. (2005). The hidden causes of surgery-resistant temporal lobe epilepsy: extratemporal or temporal plus? editorial review. Curr. Opin. Neurol. 18 (2), 125–127. 10.1097/01.wco.0000162852.22026.6f 15791141

[B55] Ryvlin P. Kahane P. Isnard J. (2001). Temporal plus epilepsies II: surgical results. Epilepsia 42 (7), 198.11240589

[B56] Saling M. M. (2009). Verbal memory in mesial temporal lobe epilepsy: beyond material specificity. Brain 132 (3), 570–582. 10.1093/brain/awp012 19251757

[B57] Schmitt F. C. Meencke H. (2020). Factors predicting 10-year seizure freedom after temporal lobe resection. Z. Für Epileptol. 33 (1), 50–61. 10.1007/s10309-019-00302-x

[B58] Schraegle W. A. Nussbaum N. L. Stefanatos A. K. (2016). List-learning and verbal memory profiles in childhood epilepsy syndromes. Epilepsy & Behav. 62, 159–165. 10.1016/j.yebeh.2016.07.021 27484747

[B59] Scoville W. B. Milner B. (1957). Loss of recent memory after bilateral hippocampal lesions. J. Neurol. Neurosurg. Psych. 20, 11–21. 10.1136/jnnp.20.1.11 PMC497229 13406589

[B60] Shamshiri E. A. Tierney T. M. Centeno M. St Pier K. Pressler R. M. Sharp D. J. (2016). Interictal activity is an important contributor to abnormal intrinsic network connectivity in pediatric focal epilepsy. Hum. Brain Mapp. 38 (1), 221–236. 10.1002/hbm.23356 27543883 PMC6866978

[B61] Signorell A. Aho K. Alfons A. Anderegg N. Aragon T. Arachchige C. (2021). DescTools: Tools for Descriptive Statistics. R Package Version 0.99.44.

[B62] Spencer S. S. (2002). Neural networks in human epilepsy: evidence of and implications for treatment. Epilepsia 43 (3), 219–227. 10.1046/j.1528-1157.2002.26901.x 11906505

[B63] Spencer S. S. Guimaraes P. Shewmon A. (1998). “Intracranial electrodes,” in Epilepsy: a comprehensive textbook. Editors Engel J. Jr Pendley T. A. (New York, NY: Lippincott-Raven), 1719–1748.

[B64] StataCorp (2021). Stata statistical software: release 17. College Station, TX: StataCorp LLC.

[B65] Stretton J. Thompson P. (2012). Frontal lobe function in temporal lobe epilepsy. Epilepsy Res. 98 (1), 1–13. 10.1016/j.eplepsyres.2011.10.009 22100147 PMC3398387

[B66] Suresh S. Sweet J. Fastenau P. S. Lüders H. Landazuri P. Miller J. (2015). Temporal lobe epilepsy in patients with nonlesional MRI and normal memory: an SEEG study. J. Neurosurg. 123 (6), 1368–1374. 10.3171/2015.1.jns141811 26207602

[B67] Tamilia E. Madsen J. R. Grant P. E. Pearl P. L. Papadelis C. (2017). Current and emerging potential of magnetoencephalography in the detection and localization of high-frequency oscillations in epilepsy. Front. Neurol. 8, 14. 10.3389/fneur.2017.00014 28194133 PMC5276819

[B68] Téllez-Zenteno J. F. Hernández Ronquillo L. Moien-Afshari F. Wiebe S. (2010). Surgical outcomes in lesional and non-lesional epilepsy: a systematic review and meta-analysis. Epilepsy Res. 89 (2-3), 310–318. 10.1016/j.eplepsyres.2010.02.007 20227852

[B69] Tombaugh T. N. Hubley A. M. (1997). The 60-item Boston naming test: norms for cognitively intact adults aged 25 to 88 years. J. Clin. Exp. Neuropsych. 19 (6), 922–932. 10.1080/01688639708403773 9524887

[B70] Tombaugh T. N. Kozak J. Rees L. (1999). Normative data stratified by age and education for two measures of verbal fluency FAS and animal naming. Arch. Clin. Neuropsych. 14 (2), 167–177. 10.1016/s0887-6177(97)00095-4 14590600

[B71] Vlooswijk M. C. G. Jansen J. F. A. Jeukens C. R. L. P. N. Marian Majoie H. J. Hofman P. A. M. de Krom M. C. T. F. M. (2011a). Memory processes and prefrontal network dysfunction in cryptogenic epilepsy. Epilepsia 52 (8), 1467–1475. 10.1111/j.1528-1167.2011.03108.x 21635235

[B72] Wang X. Q. Lang S. Y. Hong L. U. Lin M. A. Yan-ling M. A. Yang F. (2010). Changes in extratemporal integrity and cognition in temporal lobe epilepsy: a diffusion tensor imaging study. Neurol. India 58 (6), 891–899. 10.4103/0028-3886.73739 21150056

[B73] Whiteside D. M. Kealey T. Semla M. Luu H. Rice L. Basso M. R. (2015). Verbal fluency: language or executive function measure? Appl. Neuropsychol. Adult 23 (1), 29–34. 10.1080/23279095.2015.1004574 26111011

[B74] Wilson E. B. (1927). Probable inference, the law of succession, and statistical inference. J. Am. Stat. Assoc. 22 (158), 209–212. 10.1080/01621459.1927.10502953

