# Supplementary material for: The network is more important than the node: stereo-EEG evidence of neurocognitive networks in epilepsy
Source: Front Netw Physiol. 2024 Jul 24;4:1424004. doi: 10.3389/fnetp.2024.1424004 (PMC11303167; doi:10.3389/fnetp.2024.1424004)
Supplement: Supplementary file 1 [file DataSheet1.pdf]

frontiers

Supplementary Material

Presurgical Evaluation

Each patient underwent video-EEG monitoring, neuroimaging investigations and neuropsychological assessment as part of their routine care, prior to the presentation of their case at respective multidisciplinary epilepsy surgery meetings. Patients progressed to SEEG monitoring when non-invasive investigations were unable to localize a definite EZ. The placement of DIXI depth electrodes (DIXI Medical, France) for SEEG monitoring was based on anatomo-electro-clinical correlations, along with any localizing information acquired from their other non-invasive investigations. Implantations were unilateral or bilateral depending on the pre-implantation hypothesis. Each electrode was 0.8 mm in diameter, with multiple 2 mm contacts running down the length of the electrode (51 mm total exploration length). Each contact was separated by a 1.5 mm length of plastic insulation. The number of implanted electrodes differed among patients depending on their individual evaluations, however typically ranged from 8 to 18 electrodes. Following implantation, the intracerebral activity was recorded and examined for approximately 8-10 days, with continuous video recording. Cortical stimulation mapping was conducted as the last part of the evaluation to confirm the EZ and evaluate eloquent cortex.

The location of the EZ and IZ were determined based on clinical consensus following SEEG evaluation. As the EZ can only be confirmed following surgery (Rosenow & Lüders, 2001; Thivard et al., 2006), the epileptologist inferred its location based on the SOZ (which can be established based on the findings of SEEG). As such, the terms EZ and SOZ are often used interchangeably in the context of SEEG, particularly where a sample is not exclusively made up of postsurgical patients (Rosenow & Lüders, 2001). The IZ refers to any region that produced interictal activity, including sharp waves, spikes, spike-wave complexes and paroxysmal fast activity (Dinkelacker et al., 2016; Glennon et al., 2016).

Language dominance was inferred based on the findings of the IAP/Wada test, fMRI or clinical consensus based on features of language-dominant seizure onset (e.g., early language dysfunction during seizure). Where such data were not available, left-hemispheric language representation was assumed in right-handed patients only (e.g., Arrotta et al., 2022; Busch et al., 2018; Kaestner et al., 2022; Knopman et al., 2014, 2015). Left-handed or ambidextrous patients (n=4) were excluded if they had not had an IAP/Wada test or fMRI to establish language dominance.

Frontal and Temporal Regions of Interest

Frontal and temporal regions of interest (ROIs) were established prior to the collection of data. The ROIs used in this study were based on previous research investigating frontotemporal activity in SEEG patients (Bartolomei et al., 2010). Frontal ROIs included the anterior cingulate, orbitofrontal cortex, pre-SMA/SMA and/or middle/inferior frontal gyrus, while temporal ROIs were the amygdala, entorhinal cortex and/or hippocampus. The insular cortex was also explored as an ROI, however this region was not used to categorize patients across the studies, as it is highly connected to both frontal and temporal structures (Jobst et al., 2019).

Figure S1 depicts the incidence of IEDs within the specified ROIs for the interictal groups. The hippocampus/entorhinal cortex was involved in 84% of cases, followed by the amygdala (81%), orbitofrontal cortex (60%), anterior cingulate (37%), middle/inferior frontal gyrus (23%), and pre-SMA/SMA (5%).

Figure S1. Percentage Involvement of ROIs in Each Patient's IZ.

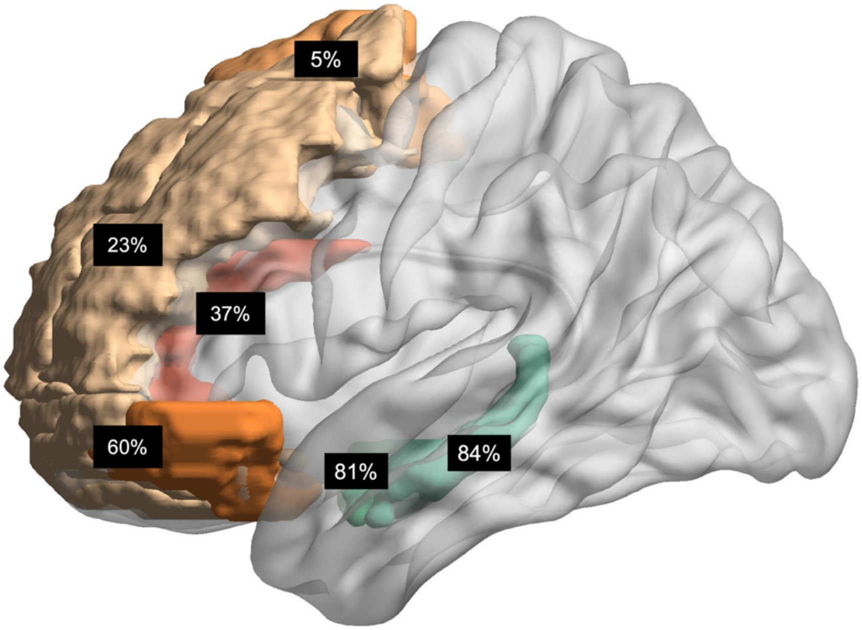

Note. ROIs are based on automated anatomical labelling (AAL-90) on a normalized brain in MNI space.

Cognitive Measures

Premorbid intelligence was estimated using the Test of Premorbid Functioning (TOPF; Pearson, 2009), National Adult Reading Test-Revised (NART-R; Blair & Spreen, 1989) or General Ability Index (GAI) where the Wechsler Adult Intelligence Scale-Fourth edition (WAIS-IV) was administered (Wechsler, 2008). Both the TOPF and NART-R require participants to read aloud a set of words with irregular pronunciations, and are used widely in clinical settings to provide an estimate of premorbid intellect, based on findings that vocabulary correlates with intellectual level achieved, but appears relatively immune to neurological decline (Bright & Van Der Linde, 2018). The GAI is used as an alternate measure of premorbid IQ, particularly when disproportionate working memory and/or processing speed deficits complicate the interpretation of a FSIQ (Wechsler, 2008). Baxendale et al. (2013) found that FSIQ may significantly underestimate premorbid intellect in people with epilepsy, as ASM polytherapy can selectively impair aspects of working memory and processing speed. As such, the GAI is thought to be a more accurate measure in an epilepsy population. Patients without an estimated premorbid intellect (n=6) were excluded from demographic analyses.

The choice of Rey Auditory Verbal Learning Test (RAVLT) measures was based on previous research investigating list-learning profiles in focal epilepsy groups (Bremm et al., 2019; McDonald et al., 2001; Schraegle et al., 2016). The variables that were used as measures of frontal lobe function were: RAVLT Trial 1 (immediate attention), RAVLT Trials 1-5 (sum of correctly recalled words across 5 trials; learning capacity), letter fluency and category fluency. The choice to include category fluency as a measure of frontal function was based on a few factors. First, while there is ongoing debate regarding whether category fluency represents a measure of temporal or frontal function (Biesbroek et al., 2016; Birn et al., 2010), studies in epilepsy, as well as frontally mediated neurodegenerative disorders, have demonstrated greater correlation between category fluency and executive tasks, compared with language tasks, despite involving components of each (Gerstenecker et al., 2013; Hermann et al., 2020). Second, significant crossover in cognitive processes (i.e., monitoring, shifting and inhibition; Miyake & Friedman, 2012; Shao et al., 2014) and regional involvement, has been observed between category and letter fluency (Thye et al., 2020; Unsworth et al., 2011). Within the literature, there is greater consensus regarding letter fluency being a measure of frontal dysfunction. In all likelihood, performance on category fluency requires input from both temporal and frontal structures, where damage to either lobe could conceivably result in impaired performance. As such, while category fluency has been included as a putative measure of frontal function, the potential input of the temporal lobe is acknowledged and will be considered when interpreting the findings of the current study.

For temporal lobe function, the RAVLT immediate retention (percentage of Trial 5 items recalled on Trial 6), delayed retention (percentage of Trial 5 items recalled after 30 minutes on Trial 7) and recognition (sum of true positives after 30 minutes) scores were examined, based on the variables included in the Schmidt metanorms (Schmidt, 1996). The Boston Naming Test (BNT) was also used to measure suspected temporal lobe function based on the impact of TLE on language (Kaestner et al., 2021; Zhao et al., 2014).

References

Arrotta, K., Reyes, A., Kaestner, E., McDonald, C. R., Hermann, B. P., Barr, W., Sarmey, N., Sundar, S. J., Kondylis, E., Najm, I., Bingaman, W., & Busch, R. M. (2022). Cognitive phenotypes in frontal lobe epilepsy. *Epilepsia, 63*(7), 1671–1681. https://doi.org/10.1111/epi.17260

Bartolomei, F., Cosandier-Rimélé, D., McGonigal, A., Aubert, S., Régis, J., Gavaret, M., Wendling, F., & Chauvel, P. (2010). From mesial temporal lobe to temporoperisylvian seizures: A quantified study of temporal lobe seizure networks. *Epilepsia*, *51*(10), 2147–2158. https://doi.org/10.1111/j.1528-1167.2010.02690.x

Baxendale, S., McGrath, K., & Thompson, P. J. (2013). Epilepsy & IQ: The clinical utility of the Wechsler Adult Intelligence Scale–Fourth Edition (WAIS–IV) indices in the neuropsychological assessment of people with epilepsy. *Journal of Clinical and Experimental Neuropsychology*, *36*(2), 137–143. https://doi.org/10.1080/13803395.2013.870535

Biesbroek, J. M., Van Zandvoort, M., Kappelle, L. J., Velthuis, B. K., Biessels, G. J., & Postma, A. (2016). Shared and distinct anatomical correlates of semantic and phonemic fluency revealed by lesion-symptom mapping in patients with ischemic stroke. *Brain Structure & Function*, *221*(4), 2123–2134. https://doi.org/10.1007/s00429-015-1033-8

Birn, R. M., Kenworthy, L., Case, L. K., Caravella, R. A., Jones, T. B., Bandettini, P. A., & Martin, A. (2010). Neural systems supporting lexical search guided by letter and semantic category

cues: A self-paced overt response fMRI study of verbal fluency. *NeuroImage*, *49*(1), 1099–1107. https://doi.org/10.1016/j.neuroimage.2009.07.036

Blair, J. R., & Spreen, O. (1989). Predicting premorbid IQ: A revision of the National Adult Reading Test. *Clinical Neuropsychologist, 3*(2), 129–136. https://doi.org/10.1080/13854048908403285

Bremm, F. J., Hendriks, M. P., Bien, C. G., & Grewe, P. (2019). Pre- and postoperative verbal memory and executive functioning in frontal versus temporal lobe epilepsy. *Epilepsy & Behavior*, *101*, 106538. https://doi.org/10.1016/j.yebeh.2019.106538

Bright, P., & Van Der Linde, I. (2018). Comparison of methods for estimating premorbid intelligence. *Neuropsychological Rehabilitation*, *30*(1), 1–14. https://doi.org/10.1080/09602011.2018.1445650

Busch, R. M., Hogue, O., Kattan, M. W., Hamberger, M., Drane, D. L., Hermann, B., Kim, M., Ferguson, L., Bingaman, W., Gonzalez-Martinez, J., Najm, I. M., & Jehi, L. (2018). Nomograms to predict naming decline after temporal lobe surgery in adults with epilepsy. *Neurology, 91*(23), e2144–e2152. https://doi.org/10.1212/WNL.0000000000006629

Dinkelacker, V., Xin, X., Baulac, M., Samson, S., & Dupont, S. (2016). Interictal epileptic discharge correlates with global and frontal cognitive dysfunction in temporal lobe epilepsy. *Epilepsy & Behavior*, *62*, 197–203. https://doi.org/10.1016/j.yebeh.2016.07.009

Gerstenecker, A., Mast, B., Duff, K., Ferman, T. J., Litvan, I., & ENGENE-PSP Study Group (2013). Executive dysfunction is the primary cognitive impairment in progressive supranuclear palsy. *Archives of Clinical Neuropsychology, 28*(2), 104–113. https://doi.org/10.1093/arclin/acs098

Glennon, J. M., Weiss-Croft, L., Harrison, S., Cross, J. H., Boyd, S. G., & Baldeweg, T. (2016). Interictal epileptiform discharges have an independent association with cognitive impairment in children with lesional epilepsy. *Epilepsia*, *57*(9), 1436–1442. https://doi.org/10.1111/epi.13479

Hermann, B., Conant, L. L., Cook, C. J., Hwang, G., Garcia-Ramos, C., Dabbs, K., Nair, V. A., Mathis, J., Bonet, C. N. R., Allen, L., Almane, D. N., Arkush, K., Birn, R., DeYoe, E. A., Felton, E., Maganti, R., Nencka, A., Raghavan, M., Shah, U., . . . Meyerand, M. E. (2020). Network, clinical and sociodemographic features of cognitive phenotypes in temporal lobe epilepsy. *NeuroImage: Clinical*, *27*, 102341. https://doi.org/10.1016/j.nicl.2020.102341

Jobst, B. C., Gonzalez-Martinez, J., Isnard, J., Kahane, P., Lacuey, N., Lahtoo, S. D., Nguyen, D. K., Wu, C., & Lado, F. (2019). The insula and its epilepsies. *Epilepsy Currents*, *19*(1), 11–21. https://doi.org/10.1177/1535759718822847

Kaestner, E., Reyes, A., Chen, A., Rao, J., Macari, A. C., Choi, J. Y., Qiu, D., Hewitt, K., Wang, Z. I., Drane, D. L., Hermann, B., Busch, R. M., Punia, V., & McDonald, C. R. (2021). Atrophy and cognitive profiles in older adults with temporal lobe epilepsy are similar to mild cognitive impairment. *Brain*, *144*(1), 236–250. https://doi.org/10.1093/brain/awaa397

Kaestner, E., Stasenko, A., Ben-Haim, S., Shih, J., Paul, B. M., & McDonald, C. R. (2022). The importance of basal-temporal white matter to pre- and post-surgical naming ability in temporal lobe epilepsy. *NeuroImage: Clinical, 34*, 102963. https://doi.org/10.1016/j.nicl.2022.102963

Knopman, A. A., Wong, C. H., Stevenson, R. J., Homewood, J., Mohamed, A., Somerville, E., Eberl, S., Wen, L., Fulham, M., & Bleasel, A. F. (2015). The relationship between neuropsychological functioning and FDG-PET hypometabolism in intractable mesial temporal lobe epilepsy. *Epilepsy & Behavior*, *44*, 136–142. https://doi.org/10.1016/j.yebeh.2015.01.023

Knopman, A. A., Wong, C., Stevenson, R. J., Homewood, J., Mohamed, A., Somerville, E., Eberl, S., Wen, L., Fulham, M., & Bleasel, A. F. (2014). The cognitive profile of occipital lobe epilepsy and the selective association of left temporal lobe hypometabolism with verbal memory impairment. *Epilepsia*, *55*(8). https://doi.org/10.1111/epi.12623

McDonald, C. R., Bauer, R. M., Grande, L., Gilmore, R. L., & Roper, S. N. (2001). The role of the frontal lobes in memory: evidence from unilateral frontal resections for relief of intractable epilepsy. *Archives of Clinical Neuropsychology*, *16*(6), 571–585. https://doi.org/10.1093/arclin/16.6.571

Miyake, A., & Friedman, N. P. (2012). The nature and organization of individual differences in executive functions. *Current Directions in Psychological Science, 21*(1), 8–14. https://doi.org/10.1177/0963721411429458

Pearson, N. C. S. (2009). *Advanced clinical solutions for WAIS-IV and WMS-IV: Administration and scoring manual.* San Antonio: The Psychological Corporation.

Rosenow, F., & Lüders, H. (2001). Presurgical evaluation of epilepsy. *Brain*, *124*(9), 1683–1700. https://doi.org/10.1093/brain/124.9.1683

Schmidt, M. (1996). *The Rey auditory verbal learning test A handbook*. Los Angeles (CA): Western Psychological Services.

Schraegle, W. A., Nussbaum, N. L., & Stefanatos, A. K. (2016). List-learning and verbal memory profiles in childhood epilepsy syndromes. *Epilepsy & Behavior*, *62*, 159–165. https://doi.org/10.1016/j.yebeh.2016.07.021

Shao, Z., Janse, E., Visser, K., & Meyer, A. S. (2014). What do verbal fluency tasks measure? Predictors of verbal fluency performance in older adults. *Frontiers in Psychology, 5*. https://doi.org/10.3389/fpsyg.2014.00772

Thivard, L., Adam, C., Hasboun, D., Clémenceau, S., Dezamis, E., Lehéricy, S., Dormont, D., Chiras, J., Baulac, M., & Dupont, S. (2006). Interictal diffusion MRI in partial epilepsies explored with intracerebral electrodes. *Brain*, *129*(2), 375–385. https://doi.org/10.1093/brain/awh709

Thye, M., Szaflarski, J. P., & Mirman, D. (2020). Shared lesion correlates of semantic and letter fluency in post-stroke aphasia. *Journal of Neuropsychology*, *15*(1), 143–150. https://doi.org/10.1111/jnp.12211

Unsworth, N., Spillers, G. J., & Brewer, G. A. (2011). Variation in verbal fluency: A latent variable analysis of clustering, switching, and overall performance. *Quarterly Journal of Experimental Psychology, 64*(3), 447–466. https://doi.org/10.1080/17470218.2010.505292

Wechsler, D. (2008). *Wechsler Adult Intelligence Scale (4th ed.).* San Antonio, TX: Pearson Assessment.

Zhao, F., Kang, H., & You, L., Rastogi, P., Venkatesh, D., & Chandra, M. (2014). Neuropsychological deficits in temporal lobe epilepsy: A comprehensive review. *Annals of Indian Academy of Neurology*, *17*(4), 374. https://doi.org/10.4103/0972-2327.144003

Table S1. Cognitive performance in frontal lobe epilepsy (FLE) and temporal lobe epilepsy (TLE) groups compared to the normative sample.

	Continuous Analyses					Categorical Analyses		
	Cognitive Domain	T-value	Mean z-score (SD)	p	r	No Imp. % (95%CI)	Mild Imp. % (95%CI)	Moderate/Severe Imp. % (95%CI)
FLE (15)	Imm Att	26	-0.57 (0.93)	.053	.50	66.7 (41.7 84.8)	20.0 (7.0, 45.2)	13.3 (3.7, 37.9)
	Learn Cap	9	-1.04 (1.05)	**.002**	.75	**53.3 (30.1, 75.2)**	6.7 (1.2, 29.8)	**40.0 (19.8, 64.2)**
	Imm Ret	35	-0.27 (0.65)	.164	.36	93.3 (70.2, 98.8)	6.7 (1.2, 29.8)	0 (0, 20.4)
	Del Ret	14	-0.54 (0.66)	**.006**	.67	80.0 (54.8, 92.9)	13.3 (3.7, 37.9)	6.7 (1.2, 29.8)
	Recog	34	-0.74 (1.54)	.139	.38	73.3 (48.0, 89.1)	6.7 (1.2, 29.8)	**20.0 (7.0, 45.2)**
	L Fluency	18	-1.14 (1.94)	**.015**	.61	**26.7 (10.9, 51.9)**	**33.3 (15.2, 58.3)**	**40.0 (19.8, 64.2)**
	C Fluency	40.5	-0.34 (1.06)	.268	.28	73.3 (48.0, 89.1)	13.3 (3.7, 37.9)	13.3 (3.7, 37.9)
	Naming	0	-2.13 (1.55)	**<.001**	.88	**20.0 (7.0, 45.2)**	20.0 (7.0, 45.2)	**60.0 (35.7, 80.2)**
TLE (33)	Imm Att	161.5	-0.29 (0.77)	**.035**	.36	84.8 (69.1, 93.3)	9.1 (3.1, 23.6)	6.1 (1.7, 19.6)
	Learn Cap	133	-0.46 (0.96)	**.007**	.46	84.8 (69.1, 93.3)	9.1 (3.1, 23.6)	6.1 (1.7, 19.6)
	Imm Ret	180	-0.36 (0.95)	.073	.31	81.8 (65.6, 91.4)	9.1 (3.1, 23.6)	9.1 (3.1, 23.6)
	Del Ret	101	-0.55 (0.93)	**<.001**	.56	75.7 (59.0, 87.1)	15.1 (6.6, 30.9)	9.1 (3.1, 23.6)
	Recog	110	-0.66 (1.31)	**.003**	.51	**69.7 (52.7, 82.6)**	12.1 (4.8, 27.3)	**18.2 (8.6, 34.4)**
	L Fluency	51.5	-0.92 (0.96)	**<.001**	.71	**48.5 (32.5, 64.8)**	**30.3 (17.4, 47.3)**	**21.2 (10.7, 37.7)**
	C Fluency	152	-0.43 (1.27)	**.020**	.40	**57.5 (40.1, 72.8)**	**36.4 (22.2, 53.4)**	6.1 (1.7, 19.6)
	Naming	20	-1.65 (1.73)	**<.001**	.81	**42.4 (27.2, 59.2)**	15.1 (6.6, 30.9)	**42.4 (27.2, 59.2)**

Note. T-value = sum of positive ranks. Bold font = $p<.05$ for continuous analyses or 95%CI does not include the expected percentage in normative sample for categorical analyses (84% no impairment; 11% mild impairment; 5% moderate/severe impairment). Imm Att = immediate attention (Trial 1); Learn Cap = learning capacity (Trials 1-5); Imm Ret = immediate retention; Del Ret = delayed retention; Recog = recognition; L Fluency = letter fluency; C Fluency = category fluency.

Table S2. Comparison of cognitive performance in frontal lobe epilepsy (FLE) and temporal lobe epilepsy (TLE) groups based on continuous data (Wilcoxon rank-sum showing Mann-Whitney U statistic).

	Seizure Onset Groups					
	FLE (*n*=15)	TLE (*n*=33)				
Cognitive domain	Rank sum	Rank sum	*U*	*z*	*p*	*r*
Imm Att	285.5	890.5	165.5	-1.83	.068	.26
Learn Cap	277	899	157	-2.01	**.044**	.29
Imm Ret	359	817	239	-0.19	.856	.03
Del Ret	350.5	825.5	230.5	-0.38	.712	.05
Recog	366.5	809.5	246.5	-0.02	.987	.00
L Fluency	298	878	178	-1.55	.124	.22
C Fluency	402	774	213	0.77	.450	.11
Naming	304	872	184	-1.41	.161	.20

Note. Bold font = $p<.05$. Imm Att = immediate attention (Trial 1); Learn Cap = learning capacity (Trials 1-5); Imm Ret = immediate retention; Del Ret = delayed retention; Recog = recognition; L Fluency = letter fluency; C Fluency = category fluency.

Table S3. Comparative proportions of patients in each severity domain by cognitive variable in frontal lobe epilepsy (FLE) and temporal lobe epilepsy (TLE) groups (results show count and percentage; p-value from Fisher's exact test).

Cognitive Domain Severity	FLE (*n*=15)	TLE (*n*=33)	*p*
Imm Att, n (%)			
No Imp.	10 (67)	28 (85)	.329
Mild Imp.	3 (20)	3 (9)	
Mod/Severe Imp.	2 (13)	2 (6)	
Learn Cap, n (%)			
No Imp.	8 (53)	28 (85)	**.016**
Mild Imp.	1 (7)	3 (9)	
Mod/Severe Imp.	6 (40)	2 (6)	
Imm Ret, n (%)			
No Imp.	14 (93)	27 (82)	.677
Mild Imp.	1 (7)	3 (9)	
Mod/Severe Imp.	0 (0)	3 (9)	
Del Ret, n (%)			
No Imp.	12 (80)	25 (76)	1.000
Mild Imp.	2 (13)	5 (15)	
Mod/Severe Imp.	1 (7)	3 (9)	
Recog, n (%)			
No Imp.	11 (73)	23 (70)	1.000
Mild Imp.	1 (7)	4 (12)	
Mod/Severe Imp.	3 (20)	6 (18)	
L Fluency, n (%)			
No Imp.	4 (27)	16 (48)	.281
Mild Imp.	5 (33)	10 (30)	
Mod/Severe Imp.	6 (40)	7 (21)	
C Fluency, n (%)			
No Imp.	11 (73)	19 (58)	.210
Mild Imp.	2 (13)	12 (36)	
Mod/Severe Imp.	2 (13)	2 (6)	
Naming, n (%)			
No Imp.	3 (20)	14 (42)	.316
Mild Imp.	3 (20)	5 (15)	
Mod/Severe Imp.	9 (60)	14 (42)	

Note. No impairment = z >-1.0; Mild impairment = -1>z>-1.64; Moderate/Severe impairment = z-score≤-1.64. Imm Att = immediate attention (Trial 1); Learn Cap = learning capacity (Trials 1-5); Imm Ret = immediate retention; Del Ret = delayed retention; Recog = recognition; L Fluency = letter fluency; C Fluency = category fluency. Bold font = p<.05.

Table S4. Cognitive performance in irritative groups compared to the normative sample.

	Cognitive Domain	T-value	Mean z-score (SD)	p	r	No Imp. % (95%CI)	Mild Imp. % (95%CI)	Moderate/Severe Imp. % (95%CI)
		Continuous Analyses				***Categorical Analyses***		
FL (5)	Imm Att	9	0.15 (1.02)	.750	.18	100.0 (56.5, 100)	0.0 (0.0, 43.4)	0.0 (0.0, 43.4)
	Learn Cap	6	-0.30 (1.27)	.812	.18	80.0 (37.5, 96.4)	0.0 (0.0, 43.4)	20.0 (3.6, 62.4)
	Imm Ret	14	0.46 (0.58)	.125	.80	100.0 (56.5, 100)	0.0 (0.0, 43.4)	0.0 (0.0, 43.4)
	Del Ret	8	0.21 (0.84)	1.00	.06	100.0 (56.5, 100)	0.0 (0.0, 43.4)	0.0 (0.0, 43.4)
	Recog	10	0.30 (0.83)	.750	.32	80.0 (37.5, 96.4)	0.0 (0.0, 43.4)	20.0 (3.6, 62.4)
	L Fluency	0	-1.30 (0.87)	.062	.90	**40.0 (11.8, 77.0)**	20.0 (3.6, 62.4)	**40.0 (11.8, 77.0)**
	C Fluency	9	0.54 (0.86)	.812	.18	80.0 (37.5, 96.4)	0.0 (0.0, 43.4)	20.0 (3.6, 62.4)
	Naming	3	-0.83 (1.07)	.312	.54	**60.0 (23.1, 88.2)**	0.0 (0.0, 43.4)	**40.0 (11.8, 77.0)**
TL (13)	Imm Att	19.5	-038 (0.75)	.067	.51	84.6 (57.8, 95.7)	7.7 (1.4, 33.3)	7.7 (1.4, 33.3)
	Learn Cap	12	-0.76 (1.15)	**.017**	.65	69.2 (42.4, 87.3)	23.1 (8.2, 50.3)	7.7 (1.4, 33.3)
	Imm Ret	33	-0.25 (1.04)	.404	.24	92.3 (66.7, 98.6)	0.0 (0.0, 22.8)	7.7 (1.4, 33.3)
	Del Ret	20	-0.73 (1.31)	.080	.49	69.2 (42.4, 87.3)	15.4 (4.3, 42.2)	15.4 (4.3, 42.2)
	Recog	19	-0.40 (0.86)	.137	.43	76.9 (49.7, 91.8)	7.7 (1.4, 33.3)	15.4 (4.3, 42.2)
	L Fluency	18.5	-0.56 (0.97)	.059	.52	61.5 (35.5, 82.3)	23.1 (8.2, 50.3)	15.4 (4.3, 42.2)
	C Fluency	44	0.07 (1.27)	.946	.03	76.9 (49.7, 91.8)	23.1 (8.2, 50.3)	0.0 (0.0, 22.8)
	Naming	12	-1.64 (2.08)	**.016**	.65	**38.5 (17.7, 64.5)**	15.4 (4.3, 42.2)	**46.1 (23.2, 70.9)**
FT (26)	Imm Att	61	-0.48 (0.79)	**.003**	.57	84.6 (66.5, 93.8)	3.8 (0.7, 18.9)	11.5 (4.0, 29.0)
	Learn Cap	59	-0.58 (0.82)	**.002**	.58	73.1 (53.9, 86.3)	15.4 (6.1 33.5)	11.5 (4.0, 29.0)
	Imm Ret	60	-0.60 (0.87)	**.002**	.57	73.1 (53.9, 86.3)	19.2 (8.5, 37.9)	7.7 (0.2, 24.1)
	Del Ret	33	-0.57 (0.63)	**<.001**	.71	73.1 (53.9, 86.3)	19.2 (8.5, 37.9)	7.7 (0.2, 24.1)
	Recog	41	-0.95 (1.43)	**<.001**	.66	**69.2 (50.0, 83.5)**	3.8 (0.7, 18.9)	**26.9 (13.7, 46.1)**
	L Fluency	34	-1.09 (1.50)	**<.001**	.70	**38.5 (22.4, 57.5)**	**26.9 (13.7, 46.1)**	**34.6 (19.4, 53.8)**
	C Fluency	62	-0.66 (1.50)	**.00**	.56	**53.8 (35.5, 71.2)**	**30.8 (16.5, 50.0)**	15.4 (0.6, 33.5)
	Naming	16	-1.61 (1.43)	**<.001**	.79	**42.3 (25.5, 61.0)**	7.7 (2.1, 24.1)	**50.0 (32.1, 67.9)**

Note. T-value = sum of positive ranks. Bold font = *p*<.05 for continuous analyses or 95%CI does not include the expected percentage in normative sample for categorical analyses (84% no impairment; 11% mild impairment; 5% moderate/severe impairment). Imm Att = immediate attention (Trial 1); Learn

Cap = learning capacity (Trials 1-5); Imm Ret = immediate retention; Del Ret = delayed retention; Recog = recognition; L Fluency = letter fluency; C Fluency = category fluency.

Table S5. Comparison of cognitive performance between irritative groups based on continuous data (Kruskal-Wallis H Test).

	Irritative Groups				
	FL (*n*=5)	TL (*n*=13)	FT (*n*=26)		
Cognitive domain	Mean z-score (*SD*)	Mean z-score (*SD*)	Mean z-score (*SD*)	*p*	H^2
Imm Att	0.15 (1.02)	-038 (0.75)	-0.48 (0.79)	.563	.02
Learn Cap	-0.30 (1.27)	-0.76 (1.15)	-0.58 (0.82)	.728	.03
Imm Ret	0.46 (0.58)	-0.25 (1.04)	-0.60 (0.87)	**.048**	.09
Del Ret	0.21 (0.84)	-0.73 (1.31)	-0.57 (0.63)	.211	.04
Recog	0.30 (0.83)	-0.40 (0.86)	-0.95 (1.43)	**.038**	.11
L Fluency	-1.30 (0.87)	-0.56 (0.97)	-1.09 (1.50)	.150	.03
C Fluency	0.54 (0.86)	0.07 (1.27)	-0.66 (1.50)	.104	.05
Naming	-0.83 (1.07)	-1.64 (2.08)	-1.61 (1.43)	.628	.02

Note. Bold font = p<.05. Imm Att = immediate attention (Trial 1); Learn Cap = learning capacity (Trials 1-5); Imm Ret = immediate retention; Del Ret = delayed retention; Recog = recognition; L Fluency = letter fluency; C Fluency = category fluency.

Table S6. Comparison of cognitive performance in irritative groups based on categorical data (count and percentage; Fisher's exact test).

Cognitive Domain Severity	FL (*n*=5)	TL (*n*=13)	FT (*n*=26)	*p*
Imm Att, n (%)				
No Imp.	5 (100)	11 (84.6)	22 (84.6)	1.000
Mild Imp.	0 (0)	1 (7.7)	1 (3.8)	
Mod/Severe Imp.	0 (0)	1 (7.7)	3 (11.5)	
Learn Cap, n (%)				
No Imp.	4 (80)	9 (69.2)	19 (73.1)	.831
Mild Imp.	0 (0)	3 (23.1)	4 (15.4)	
Mod/Severe Imp.	1 (20)	1 (7.7)	3 (11.5)	
Imm Ret, n (%)				
No Imp.	5 (100)	12 (92.3)	19 (73.1)	.410
Mild Imp.	0 (0)	0 (0)	5 (19.2)	
Mod/Severe Imp.	0 (0)	1 (7.7)	2 (7.6)	
Del Ret, n (%)				
No Imp.	5 (100)	9 (69.2)	19 (73.1)	.823
Mild Imp.	0 (0)	2 (15.4)	5 (19.2)	
Mod/Severe Imp.	0 (0)	2 (15.4)	2 (7.6)	
Recog, n (%)				
No Imp.	4 (80)	10 (76.9)	18 (69.2)	.461
Mild Imp.	1 (20)	1 (7.7)	1 (3.8)	
Mod/Severe Imp.	0 (0)	2 (15.4)	7 (26.9)	
L Fluency, n (%)				
No Imp.	2 (40)	8 (61.5)	10 (38.5)	.702
Mild Imp.	1 (20)	3 (23.1)	7 (26.9)	
Mod/Severe Imp.	2 (40)	2 (15.4)	9 (34.6)	
C Fluency, n (%)				
No Imp.	4 (80)	10 (76.9)	14 (53.8)	.551
Mild Imp.	1 (20)	3 (23.1)	8 (30.8)	
Mod/Severe Imp.	0 (0)	0 (0)	4 (15.4)	
Naming, n (%)				
No Imp.	3 (60)	5 (38.5)	11 (42.3)	.881
Mild Imp.	0 (0)	2 (15.4)	2 (7.7)	
Mod/Severe Imp.	2 (40)	6 (46.1)	13 (50.0)	

Note. No impairment = z >-1.0; Mild impairment = -1>z>-1.64; Moderate/Severe impairment = z-score≤-1.64. Imm Att = immediate attention (Trial 1); Learn Cap = learning capacity (Trials 1-5); Imm Ret = immediate retention; Del Ret = delayed retention; Recog = recognition; L Fluency = letter fluency; C Fluency = category fluency. Bold font = p<.05.

Table S7. Cognitive performance in subdivided frontotemporal groups compared to the normative sample (z=0; Wilcoxon signed-rank).

		Continuous Analyses				***Categorical Analyses***		
	Cognitive Domain	T-value	Mean z-score (SD)	p	r	No Imp. % (95%CI)	Mild Imp. % (95%CI)	Moderate/Severe Imp. % (95%CI)
TL-F (17)	Imm Att	42	-0.28 (0.81)	.111	.40	88.2 (65.7, 96.7)	5.9 (1.0, 27.0)	5.9 (1.0, 27.0)
	Learn Cap	43	-0.34 (0.80)	.117	.38	88.2 (65.7, 96.7)	5.9 (1.0, 27.0)	5.9 (1.0, 27.0)
	Imm Ret	32	-0.57 (0.96)	**.034**	.51	76.5 (52.7, 90.4)	11.8 (3.3, 34.3)	11.8 (3.3, 34.3)
	Del Ret	22	-0.47 (0.60)	**.008**	.63	76.5 (52.7, 90.4)	17.6 (6.2, 41.0)	5.9 (1.0, 27.0)
	Recog	20	-0.92 (1.58)	**.010**	.62	70.6 (46.9, 86.7)	5.9 (1.0, 27.0)	**23.5 (9.5, 47.3)**
	L Fluency	3	-1.23 (0.94)	**<.001**	.84	**41.2 (21.6, 64.0)**	**29.4 (13.3, 53.1)**	**29.4 (13.3, 53.1)**
	C Fluency	27	-0.62 (1.31)	**.017**	.57	52.9 (31.0, 73.8)	**35.3 (17.3, 58.7)**	11.8 (3.3, 34.3)
	Naming	13.5	-1.33 (1.51)	**.001**	.72	**58.8 (36.0, 78.4)**	0.0 (0.0, 18.4)	**41.2 (21.6, 64.0)**
FL-T (9)	Imm Att	1	-0.84 (0.65)	**.008**	.86	**44.4 (18.9, 73.3)**	**33.3 (12.1, 64.6)**	**22.2 (6.3, 54.7)**
	Learn Cap	1	-1.01 (0.70)	**.008**	.85	**44.4 (18.9, 73.3)**	**33.3 (12.1, 64.6)**	**22.2 (6.3, 54.7)**
	Imm Ret	5	-0.68 (0.74)	**.039**	.69	66.7 (35.4, 87.9)	**33.3 (12.1, 64.6)**	0.0 (0.0, 29.9)
	Del Ret	2	-0.76 (0.66)	**.012**	.81	66.7 (35.4, 87.9)	22.2 (6.3, 54.7)	11.1 (2.0, 43.5)
	Recog	4	-0.99 (1.17)	**.027**	.73	66.7 (35.4, 87.9)	0.0 (0.0, 29.9)	**33.3 (12.1, 64.6)**
	L Fluency	11	-0.81 (2.27)	.195	.45	**33.3 (12.1, 64.6)**	22.2 (6.3, 54.7)	**44.4 (18.9, 73.3)**
	C Fluency	8	-0.72 (1.05)	.098	.57	**55.5 (26.7, 81.1)**	22.2 (6.3, 54.7)	**22.2 (6.3, 54.7)**
	Naming	0	-2.14 (1.17)	**.004**	.89	**11.1 (2.0, 43.5)**	22.2 (6.3, 54.7)	**66.7 (35.4, 87.9)**

Note. T-value = sum of positive ranks. Bold font = $p<.05$ for continuous analyses or 95%CI does not include the expected percentage in normative sample for categorical analyses (84% no impairment; 11% mild impairment; 5% moderate/severe impairment). Imm Att = immediate attention (Trial 1); Learn Cap = learning capacity (Trials 1-5); Imm Ret = immediate retention; Del Ret = delayed retention; Recog = recognition; L Fluency = letter fluency; C Fluency = category fluency.

Table S8. Comparison of cognitive performance between patients with isolated temporal irritative networks (TL) and temporal to frontal irritative networks (TL-F) based on continuous data (Wilcoxon rank-sum test showing Mann-Whitney U Statistic).

	Irritative Groups					
	TL (n=13)	*TL-F (n=17)*				
Cognitive domain	Rank sum	Rank sum	*U*	*z*	*p*	r
Imm Att	195	270	104	-0.27	.796	.04
Learn Cap	170	295	79	-1.32	.194	.24
Imm Ret	222.5	242.5	89.5	0.88	.391	.16
Del Ret	203	262	109	0.06	.967	.01
Recog	225	240	87	0.99	.331	.18
L Fluency	240	225	72	1.61	.110	.29
C Fluency	236.5	228.5	75.5	1.47	.147	.27
Naming	201	264	110	-0.02	.992	.00

Note. Imm Att = immediate attention (Trial 1); Learn Cap = learning capacity (Trials 1-5); Imm Ret = immediate retention; Del Ret = delayed retention; Recog = recognition; L Fluency = letter fluency; C Fluency = category fluency.

Table S9. Comparison of cognitive performance between patients with isolated temporal irritative networks (TL) and temporal to frontal irritative networks (TL-F) based on categorical data (count and percentage; Fisher's exact test).

Cognitive Domain Severity	TL (*n*=13)	TL-F (*n*=17)	*p*
Imm Att, n (%)			
No Imp.	11 (84.6)	15 (88.2)	1.000
Mild Imp.	1 (7.7)	1 (5.9)	
Mod/Severe Imp.	1 (7.7)	1 (5.9)	
Learn Cap, n (%)			
No Imp.	9 (69.2)	15 (88.2)	.421
Mild Imp.	3 (23.1)	1 (5.9)	
Mod/Severe Imp.	1 (7.7)	1 (5.9)	
Imm Ret, n (%)			
No Imp.	12 (92.3)	13 (76.5)	.613
Mild Imp.	0 (0)	2 (11.8)	
Mod/Severe Imp.	1 (7.7)	2 (11.8)	
Del Ret, n (%)			
No Imp.	9 (69.2)	13 (76.5)	.838
Mild Imp.	2 (15.4)	3 (17.6)	
Mod/Severe Imp.	2 (15.4)	1 (5.9)	
Recog, n (%)			
No Imp.	10 (76.9)	12 (70.6)	.834
Mild Imp.	1 (7.7)	1 (5.9)	
Mod/Severe Imp.	2 (15.4)	4 (23.5)	
L Fluency, n (%)			
No Imp.	8 (61.5)	7 (41.2)	.631
Mild Imp.	3 (23.1)	5 (29.4)	
Mod/Severe Imp.	2 (15.4)	5 (29.4)	
C Fluency, n (%)			
No Imp.	10 (76.9)	9 (52.9)	.429
Mild Imp.	3 (23.1)	6 (35.3)	
Mod/Severe Imp.	0 (0)	2 (11.8)	
Naming, n (%)			
No Imp.	5 (38.5)	10 (58.8)	.269
Mild Imp.	2 (15.4)	0 (15)	
Mod/Severe Imp.	6 (46.1)	7 (41.2)	

Note. No impairment = z >-1.0; Mild impairment = -1>z>-1.64; Moderate/Severe impairment = z-score≤-1.64. Imm Att = immediate attention (Trial 1); Learn Cap = learning capacity (Trials 1-5); Imm Ret = immediate retention; Del Ret = delayed retention; Recog = recognition; L Fluency = letter fluency; C Fluency = category fluency. Bold font = p<.05.

Table S10. Comparison of cognitive performance between patients with isolated frontal irritative networks (FL) and frontal to temporal irritative networks (FL-T) based on continuous data (Wilcoxon rank-sum test showing Mann-Whitney U Statistic).

	Irritative Groups					
	FL (n=5)	*FL-T (n=9)*				
Cognitive domain	Rank sum	Rank sum	*U*	*z*	*p*	r
Imm Att	51.5	53.5	8.5	1.89	.067	.50
Learn Cap	46	59	14	1.33	.285	.35
Imm Ret	54	51	6	2.21	**.027**	.59
Del Ret	52	53	8	1.93	.059	.52
Recog	54	51	6	2.23	**.027**	.60
L Fluency	40.5	64.5	19.5	0.40	.723	.11
C Fluency	47	58	13	1.27	.240	.34
Naming	52	53	8	1.93	.059	.52

Note. Bold font = $p<.05$. Imm Att = immediate attention (Trial 1); Learn Cap = learning capacity (Trials 1-5); Imm Ret = immediate retention; Del Ret = delayed retention; Recog = recognition; L Fluency = letter fluency; C Fluency = category fluency.

Table S11. Comparison of cognitive performance between patients with isolated frontal irritative networks (FL) and frontal to temporal irritative networks (FL-T) based on categorical data (count and percentage; Fisher's exact test).

Cognitive Domain Severity	FL (*n*=5)	FL-T (*n*=9)	*p*
Imm Att, n (%)			
No Imp.	5 (100)	7 (77.8)	.505
Mild Imp.	0 (0)	0 (0)	
Mod/Severe Imp.	0 (0)	2 (22.2)	
Learn Cap, n (%)			
No Imp.	4 (80)	4 (44.4)	.497
Mild Imp.	0 (0)	3 (33.3)	
Mod/Severe Imp.	1 (20)	2 (22.2)	
Imm Ret, n (%)			
No Imp.	5 (100)	6 (66.7)	.258
Mild Imp.	0 (0)	3 (33.3)	
Mod/Severe Imp.	0 (0)	0 (0)	
Del Ret, n (%)			
No Imp.	5 (100)	6 (66.7)	.670
Mild Imp.	0 (0)	2 (22.2)	
Mod/Severe Imp.	0 (0)	1 (11.1)	
Recog, n (%)			
No Imp.	4 (80)	6 (66.7)	.200
Mild Imp.	1 (20)	0 (0)	
Mod/Severe Imp.	0 (0)	3 (33.3)	
L Fluency, n (%)			
No Imp.	2 (40)	3 (33.3)	1.000
Mild Imp.	1 (20)	2 (22.2)	
Mod/Severe Imp.	2 (40)	4 (44.4)	
C Fluency, n (%)			
No Imp.	4 (80)	5 (55.5)	.748
Mild Imp.	1 (20)	2 (22.2)	
Mod/Severe Imp.	0 (0)	2 (22.2)	
Naming, n (%)			
No Imp.	3 (60)	1 (11.1)	.231
Mild Imp.	0 (0)	2 (22.2)	
Mod/Severe Imp.	2 (40)	6 (66.7)	

Note. No impairment = z >-1.0; Mild impairment = -1>z>-1.64; Moderate/Severe impairment = z-score≤-1.64. Imm Att = immediate attention (Trial 1); Learn Cap = learning capacity (Trials 1-5); Imm Ret = immediate retention; Del Ret = delayed retention; Recog = recognition; L Fluency = letter fluency; C Fluency = category fluency. Bold font = $p<.05$.

Table S12. Comparison of cognitive performance between subdivided frontotemporal groups based on continuous data (Wilcoxon rank-sum test showing Mann-Whitney U Statistic).

	Epilepsy Groups					
	TL-F (n=17)	*FL-T (n=9)*				
Cognitive domain	Rank sum	Rank sum	*U*	*z*	*p*	r
Imm Att	271	80	35	2.24	**.023**	44
Learn Cap	272.5	78.5	33.5	2.32	**.019**	.45
Imm Ret	240	111	66	0.57	.587	.11
Del Ret	249	102	57	1.05	.312	.21
Recog	242	109	64	0.68	.515	.13
L Fluency	236.5	114.5	69.5	0.38	.721	.07
C Fluency	228.5	122.5	75.5	-0.05	.968	.01
Naming	254.5	96.5	51.5	1.35	.186	.26

Note. Bold font = $p<.05$. Imm Att = immediate attention (Trial 1); Learn Cap = learning capacity (Trials 1-5); Imm Ret = immediate retention; Del Ret = delayed retention; Recog = recognition; L Fluency = letter fluency; C Fluency = category fluency.

Table S13. Comparison of cognitive performance in subdivided frontotemporal groups based on categorical data (count and percentage; Fisher's exact test).

Cognitive Domain Severity	TL-F (*n*=17)	FL-T (*n*=9)	*p*
Imm Att, n (%)			
No Imp.	15 (88.2)	7 (77.8)	.693
Mild Imp.	1 (5.9)	0 (0)	
Mod/Severe Imp.	1 (5.9)	2 (22.2)	
Learn Cap, n (%)			
No Imp.	15 (88.2)	4 (44.4)	*.056*
Mild Imp.	1 (5.9)	3 (33.3)	
Mod/Severe Imp.	1 (5.9)	2 (22.2)	
Imm Ret, n (%)			
No Imp.	13 (76.5)	6 (66.7)	.383
Mild Imp.	2 (11.8)	3 (33.3)	
Mod/Severe Imp.	2 (11.8)	0 (0)	
Del Ret, n (%)			
No Imp.	13 (76.5)	6 (66.7)	1.000
Mild Imp.	3 (17.6)	2 (22.2)	
Mod/Severe Imp.	1 (5.9)	1 (11.1)	
Recog, n (%)			
No Imp.	12 (70.6)	6 (66.7)	.786
Mild Imp.	1 (5.9)	0 (0)	
Mod/Severe Imp.	4 (23.5)	3 (33.3)	
L Fluency, n (%)			
No Imp.	7 (41.2)	3 (33.3)	.769
Mild Imp.	5 (29.4)	2 (22.2)	
Mod/Severe Imp.	5 (29.4)	4 (44.4)	
C Fluency, n (%)			
No Imp.	9 (52.9)	5 (55.5)	.856
Mild Imp.	6 (35.3)	2 (22.2)	
Mod/Severe Imp.	2 (11.8)	2 (22.2)	
Naming, n (%)			
No Imp.	10 (58.8)	1 (11.1)	**.018**
Mild Imp.	0 (15)	2 (22.2)	
Mod/Severe Imp.	7 (41.2)	6 (66.7)	

Note. No impairment = z >-1.0; Mild impairment = -1>z>-1.64; Moderate/Severe impairment = z-score≤-1.64. Imm Att = immediate attention (Trial 1); Learn Cap = learning capacity (Trials 1-5); Imm Ret = immediate retention; Del Ret = delayed retention; Recog = recognition; L Fluency = letter fluency; C Fluency = category fluency. Bold font = *p*<.05; *Italicised* = approaching significance.
